# Swap errors in visual working memory are fully explained by cue-feature variability

**DOI:** 10.1016/j.cogpsych.2022.101493

**Published:** 2022-06-28

**Authors:** Jessica MV McMaster, Ivan Tomić, Sebastian Schneegans, Paul M Bays

**Affiliations:** Department of Psychology, University of Cambridge, Cambridge, UK

**Keywords:** swap error, intrusion error, visual working memory, short-term memory, feature binding, cued recall

## Abstract

In cue-based recall from working memory, incorrectly reporting features of an uncued item may be referred to as a “swap” error. One account of these errors ascribes them to variability in memory for the cue features leading to erroneous selection of a non-target item, especially if it is similar to the target in the cue-feature dimension. However, alternative accounts of swap errors include cue-independent misbinding, and strategic guessing when the cued item is not in memory. Here we investigated the cause of swap errors by manipulating the variability with which either cue or report features (orientations in Exp 1; motion directions in Exp 2) were encoded. We found that swap errors increased with increasing variability in memory for the cue features, and their changing frequency could be quantitatively predicted based on recall variability when the same feature was used for report. These results are inconsistent with the hypothesis that swaps are a strategic response to forgotten items, and suggest that swap errors could be wholly accounted for by confusions due to cue-dimension variability. In a third experiment we examined whether spatial configuration of memory arrays in tasks with spatial cueing has an influence on swap error frequency. We observed a specific tendency to make swap errors to non-targets located precisely opposite to the cued location, suggesting that stimulus positions are partially encoded in a non-metric format.

## 1 Introduction

Short-term memory for a visual scene typically comprises the storage of multiple items, each defined by a combination of visual features such as colour, shape and orientation. Beyond storing these individual features, the memory system must also be capable of maintaining the specific conjunctions between the features that belong to the same item. The mechanism of feature binding in visual working memory (VWM) continues to be an area of active research, and various aspects of it remain contentious (for a recent review, see Schneegans & Bays, 2019).

One central question that is still a topic of debate is whether VWM representations inherently contain binding information, or whether bindings are stored independently of memory for individual features. The latter view was supported by the results of various change detection tasks. In a study by Stefurak and Boynton (1986), participants’ performance was worse when they had to decide whether a single test stimulus matched a specific color-shape combination seen in a memory array, compared to a task condition in which they only needed to detect a change to a novel color or shape (see also Treisman, Sykes, & Gelade, 1977). Based on observed change detection performance across various task conditions, Wheeler and Treisman (2002) proposed that binding is selectively stored if task-relevant, but that its storage is dependent on sustained attention, and that joint object representations break down into their constituent features if attention is withdrawn. However, the comparison of different change detection tasks does not reveal at which point response errors arise, and the observed performance differences may also be explained as a consequence of the additional computations required in tasks that involve comparisons of feature conjunctions.

**Figure 1:**
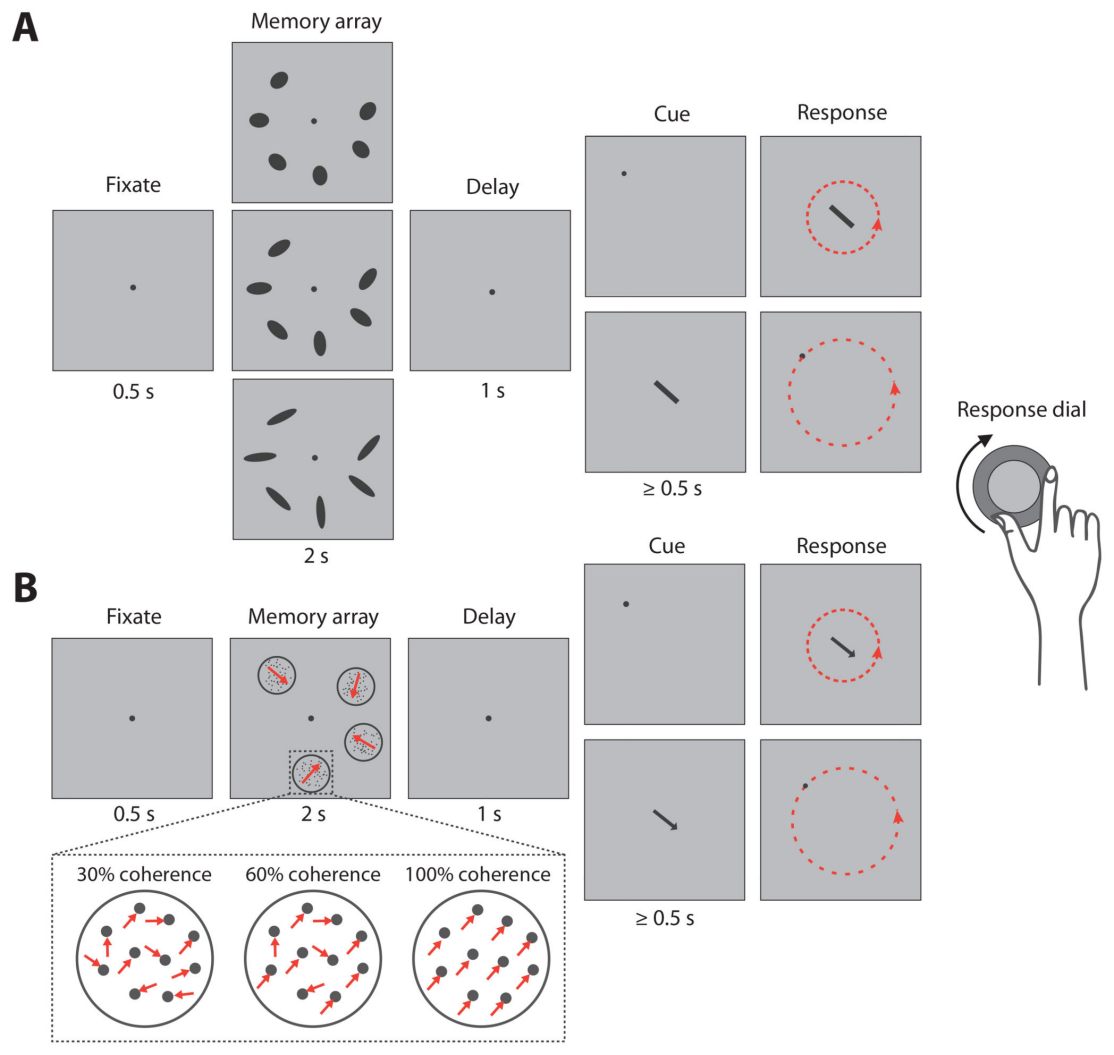
Experimental tasks. **(A)** In Experiment 1, a stimulus array was presented on each trial consisting of six oriented ellipses with one of three levels of elongation (top to bottom: low, medium and high). After a delay, participants were cued with the location or orientation of one item from the preceding array, and used a response dial to report the other feature of the same item. **(B)** The task in Experiment 2 was similar except that each memory array consisted of four motion stimuli with different directions of motion, at one of three levels of motion coherence. The location or motion direction of one item was subsequently cued and participants reported the other feature of the same item. Displays are not to scale.

The application of cued recall tasks and continuous measures of recall errors in VWM studies has led to significant advancements in our understanding of feature binding. Cued recall tasks usually involve the presentation of an array of visual items, each combining two or more features, e.g. a colour and a location. After a delay, a cue is presented consisting of one feature of one item (the target), and participants are instructed to report a second feature of that item on a continuous scale (examples in Fig. 1). Tasks involving analogue responses, rather than the binary outcomes of change detection tasks, allow for the estimation of error distributions along the reported feature dimension. This has led to an increased appreciation that the quality or precision of working memory representations can vary substantially, beyond the binary distinction between memorized and forgotten items (Ma, Husain, & Bays, 2014).

Importantly, cued recall tasks also inherently test memory for feature binding, since the presented response cue must be used to retrieve the associated report feature of the same item. This creates multiple opportunities for errors both in memory for the cue feature and memory for the report feature to affect the response. It has been observed in previous studies that response values are not only dispersed around the report feature of the target item, but are often also clustered around the feature values of other, non-target items from the memory array (Bays, Catalao, & Husain, 2009; Oberauer & Lin, 2017; Schneegans & Bays, 2017). Reporting the feature value of a non-target item is often referred to as a “swap” error, as the feature of a non-target item is “swapped” in for the target feature. Furthermore, as swap errors reflect a failure in retrieving the correct item from memory when cued with one of the item’s features, the mechanisms underlying swap errors may provide important insight into feature binding.

A number of models of VWM have incorporated feature binding within memory for single features to explain error distributions in cued recall tasks (Oberauer & Lin, 2017; Schneegans & Bays, 2017; Swan & Wyble, 2014). Both the neural binding model of Schneegans and Bays (2017) and the interference model of Oberauer and Lin (2017) successfully reproduce swap errors seen in the behavioral data on the basis of imprecise memory for the cue features of the sample items. Due to this imprecision, the given retrieval cue cannot unambiguously identify the target item, and the report feature of a non-target item may be reported, especially if the target and non-target item have similar cue feature values. We will refer to this as the feature variability account of swap errors. Importantly, in this account, the incorrect item is retrieved from memory despite the bindings between features of each item remaining intact.

The feature variability account is supported by the finding that the proportion of swap errors in a cued recall task depends on which feature is used as the memory cue, with swap errors being more frequent when location is reported using a color cue compared to when color is reported using a location cue (Rajsic, Swan, Wilson, & Pratt, 2017; Rajsic & Wilson, 2014). If swap errors were caused only by the loss of binding information, then the proportion of swap errors should be equal irrespective of the feature used as the cue. The link between swap errors and the cue-feature dimension is further supported by studies using spatial cues that have found that non-target items more similar to the cue are more likely to be the subject of swap errors than those with more dissimilar cue features (Bays, 2016a; Emrich & Ferber, 2012; Rerko, Oberauer, & Lin, 2014; Souza, Rerko, Lin, & Oberauer, 2014).

While these results strongly support cue-feature variability as one cause of swap errors, it has been debated whether it is the only or even the principal cause. The neural binding model (Schneegans & Bays, 2017) assumes that the feature combinations of all sample items are encoded in a conjunctive population code. At retrieval, cue and report features of all items are decoded from the noisy neural activity, the item whose decoded cue feature value is closest to the retrieval cue is selected, and the corresponding report feature produced as a response. Due to imprecision in decoding, the cue feature of a non-target item may be judged to be closest to the given cue, leading to a swap error. This process is consistent with the feature variability account, and is the sole source of swap errors within the neural binding model.

The interference model (Oberauer & Lin, 2017) similarly assumes a cue-dependent source of swap errors, in which the retrieval cue activates the report feature value of non-target items to a certain degree due to imprecision in memory for the cue features. However, this model additionally assumes a cue-*independent* source of swap errors. These cue-independent swaps are attributed to uniform background noise in the cue feature space that provides equal activation to all features in memory. Parameter estimates obtained by fitting this model to data have indicated the cue-independent source makes a non-zero contribution to swap frequency in both cued recall (Oberauer & Lin, 2017) and change detection tasks (Lin & Oberauer, 2022).

Swap errors have also been ascribed by some authors to an informed guessing strategy (Huang, 2019; Pratte, 2019). For example, Pratte (2019) proposed that, in addition to swaps caused by cue-feature variability, participants might sometimes be presented with a cue corresponding to an item that was not in memory at all, and that in this case a viable strategy could be to respond with the report feature of a different item that was in memory. Depending on how this item was chosen, this mechanism could also be a source of cue-independent swaps.

In addition to these proposals, any swap errors caused by loss of binding information, separately from memory for the individual features, would also be expected to occur independently of cue similarity. On the other hand, if swap errors can be fully accounted for by variability in the cue-feature dimension, this could be considered evidence against separate memory storage of feature binding, on the assumption that such storage would sometimes fail.

The main aims of the present study were: first, to provide a direct test of the hypothesized causal connection between error in recall of cue-features and swap frequency, by manipulating memory variability in the cue-feature dimension; and second, to determine whether cue-feature variability provides a sufficient explanation for swap errors, or if there is some proportion of swaps that cannot be explained by this mechanism. We used cued recall tasks in which spatial location acted either as the cue or report feature dimension, while we manipulated variability in the other feature dimension. Because angular location memory has natively high precision (Schneegans & Bays, 2017), we reasoned that in combination with large minimum separations between items, it could be used as a reliable cue, resulting in vanishingly rare swap errors. In contrast, manipulation of memory variability for the other feature was expected to affect the utility of that feature as a retrieval cue, resulting in a gradient of swap errors consistent with induced memory variability. In Experiment 1, variability in memory for orientation was manipulated using ellipse stimuli with differing elongations. In Experiment 2, memory variability for motion direction was altered using random dot kinematogram (RDK) stimuli with varying motion coherences. Similar stimulus manipulations have been used in previous VWM studies for different but related purposes (Ester, Ho, Brown, & Serences, 2014; Keshvari, van den Berg, & Ma, 2012).

Across both experiments we found an increase in swap errors as variability in memory for the cue feature was increased, as predicted by the feature variability account. To test whether the increase in memory variability could fully account for the observed pattern of swap errors, we employed a Monte Carlo simulation. Based on the observed recall errors for the non-spatial feature when cued with location, this simulation accurately predicted how frequently a non-target location would be selected for report when the same non-spatial feature was used as a cue. These results indicate that swap errors can be fully explained by confusions in identifying the cued item due to variability in the cue-feature dimension.

The results of the non-parametric simulation provide the strongest test of our main hypothesis, that the feature variability account can fully explain swap errors. However, to also assess a concrete implementation of the feature variability account that makes detailed predictions for error distributions, we fit the behavioral data with a version of the neural binding model of Schneegans and Bays (2017). This model allows for stochastic variation in memory precision including the possibility that features are retrieved with zero precision, but does not implement any form of strategic guessing. The fit of the neural binding model was compared with two variants of the interference model (Oberauer & Lin, 2017), the full model and a partial model with the cue-independent activation parameter fixed at zero. The neural binding model was consistently a better fit to the data than both variants of the interference model. Furthermore, the neural binding model produced close quantitative fits to the pattern of swap errors on all trials, across changes in ellipse elongation and RDK coherence. It also made a prediction of lower subjective confidence in swap than non-swap responses that is consistent with previous observations.

One methodological difference that has been highlighted as a potential influence on swap errors (Brady & Störmer, 2020; Schurgin, Wixted, & Brady, 2020) is between tasks in which locations of items are selected randomly from a continuous range, as in our Experiments 1 and 2, and experiments where they were selected from a fixed set of locations, as was the case in the experiments providing evidence for the cue-independent source of swap errors within the interference model (Oberauer & Lin, 2017). In Experiment 3 we directly compared these designs. While the results did not support a cue-independent source of swap errors or an effect of design on overall swap frequency, they did reveal a specific tendency to make swap errors to items located diametrically opposite to the cued location in the circular array. We argue that these errors could result from item positions being encoded in a partly non-metric format, in which diametrically opposing positions (180° separation) were more confusable than other distant locations (e.g. 120° apart).

## 2 Experiment 1

Experiment 1 was a cued recall task designed to determine whether changes in memory variability for the cue feature resulted in corresponding changes in swap error frequency. Participants were presented with arrays of ellipse stimuli with different locations and orientations. The precision with which orientations were encoded was manipulated by varying the elongation of the ellipses across trials. Recall was tested either for the orientation of an item corresponding to a cued location, or the location of an item indicated by a cued orientation.

### 2.1 Methods

#### 2.1.1 Experimental protocol

Ten participants (aged 21–31 years; six male, four female) completed a cued recall task testing their memory for orientation and location. The study was approved by the Cambridge Psychology Research Ethics Committee. All participants reported normal or corrected-to-normal visual acuity and gave informed consent in accordance with the Declaration of Helsinki. Stimuli were presented on a 27-inch LCD monitor (ASUS PG279) with a refresh rate of 144 Hz. Participants sat with their head supported by a forehead and chin rest and viewed the monitor at a distance of 60 cm. Eye position was monitored on-line at 1000 Hz using an infrared eye tracker (Eyelink 1000 Plus, SR Research).

Each trial of the task (illustrated in Fig. 1A) began with the presentation of a dark grey central fixation dot (diameter, 0.25° of visual angle) against a lighter grey background. Once a stable fixation was recorded within 3° of the dot, a memory array consisting of six ellipses was presented for 2 s. The elongation (eccentricity) of the ellipses was constant within a trial but varied between trials. Three eccentricities were used: 0.71, 0.86 and 0.97 (hereafter referred to as low, medium and high elongation, respectively), defined as $\sqrt{1-\frac{b^{2}}{a^{2}}}$, where a and b specify the ellipse’s semi-major axis and semi-minor axis. The area of the ellipses was held constant at 1.6 squared degrees of visual angle across changes in elongation, with the major axis of the ellipse varying from 1.7° of visual angle (low elongation) to 3° (high elongation). The center of each ellipse was positioned on an invisible circle with a radius of 6° centered on the fixation dot. The location of each ellipse was chosen at random, with a minimum separation between ellipse centers of 30° on the circle. The ellipses’ orientations were also chosen at random, with a minimum separation of 15° between the orientations of different ellipses (considering the space of unique ellipse orientations covers only 180°, a 15° separation was chosen to match the 30° used for location).

After the presentation of the memory array, the fixation dot was shown for a further 1 s followed by the cue display. In the orientation-report condition, the cue was a dark grey dot (diameter, 0.25° of visual angle) presented at a location corresponding to one of the six ellipses in the previous display that was randomly selected as the target item. Participants were instructed to begin turning a response dial (PowerMate USB Multimedia Controller, Griffin Technology) once they were ready to respond with the orientation of the target ellipse. Once participants began their response, the cue display was replaced with a central dark grey line (length 2°, width 0.25°) which participants freely rotated using the dial until it matched the remembered orientation of the target. Responses were not timed, and participants were instructed to be as precise as possible. In the location-report condition, participants were cued with a central line stimulus (as described above) with orientation matching the ellipse randomly selected as the target, and used the response dial to move a dot (as described above) around the invisible circle until it matched the remembered location of the target. Any trial on which gaze deviated > 3° from the central dot, before the cue display, was aborted and restarted with new feature values. Each participant completed 12 blocks (8 in the location-report condition, 4 orientation-report, in a randomized order), with each block consisting of 12 trials in each of the three elongation conditions, randomly interleaved. The 12 blocks were completed across two one-hour testing sessions. We dedicated a larger proportion of blocks to the location-report condition because this was the critical condition for testing our main hypothesis.

### 2.2 Analysis

#### 2.2.1 Response error distributions

Stimulus and response values in each feature dimension were analyzed and are reported with respect to a circular space (−*π* to *π* radians) spanning the full range of possible feature values. This range corresponded to 360° of angular location, and 180° of orientation. Recall error was calculated, for each trial, as the angular deviation between the participant’s response and the target feature value. The circular standard deviation (Fisher, 1995) of the response error was used as a measure of recall variability. Statistical hypothesis testing was based on Bayesian (using JASP, 2019) and frequentist t-tests.

In order to evaluate the influence of non-targets on recall estimates, we calculated the deviation of each response from the report feature values of each of the non-target items in the memory array, and generated histogram estimates based on pooling these deviations over trials and non-targets. Due to the minimum feature separation imposed when generating the memory arrays, the distribution of deviations expected in the absence of swap errors and any other effects of non-target items is not uniform. To see this, consider a case in which all responses are tightly clustered around the true target value. If there is a minimum separation between target and non-target feature values, all responses will also have nearly the same separation from the non-target feature values, resulting in a central dip in the distribution of response deviations from non-target values. A minimum separation between the feature values of different non-target items within a trial will further modulate the expected distribution.

We estimated the expected distribution in the absence of non-target effects using a randomization method. We removed the report feature value of a single non-target item from each trial, and replaced it with another randomly-selected value that still respected the minimum separation constraints for that particular feature dimension. Then we calculated the response deviation from the newly inserted non-target feature value (which cannot have influenced the actual response), and repeated this over 1000 iterations for each trial to obtain an expected histogram of non-target deviations. This expected histogram was subtracted from the observed histogram of response deviations to produce plots in which values consistently different from zero could be interpreted as evidence for an influence of non-targets. The same method was also used to determine an expected mean absolute deviation (MAD) of responses from non-target features, which we compared with the observed values.

#### 2.2.2 Estimating swap frequencies

Non-target responses were of particular interest in the location-report condition, where we expected the manipulation of ellipse elongation to affect the proportion of swap errors. To obtain a non-parametric estimate of swap frequency in this condition, we classified a response as a swap error if the reported location was closer to the location of one non-target item than to the true target location. Given the relatively large minimum separation between items and the precision of location memory observed in comparable previous studies (Schneegans & Bays, 2017), we expected that this simple heuristic can produce reliable estimates of swap error frequencies.

We further validated the estimates obtained from the nearest-item heuristic by comparison with two established methods of estimating swap frequencies: the mixture model method (Bays et al., 2009) and the resultant vector method of Bays (2016a). The mixture model method describes the distribution of responses as a probabilistic mixture of von Mises (circular normal) distributions centered on the target and non-target features, and a uniform distribution. This method uses maximum likelihood optimisation to estimate the mixing weights of each component distribution. Importantly, the accuracy of this method’s estimates depends on the correct specification of the response error distribution. In contrast, the resultant vector method is a non-parametric method that does not make assumptions about the form of the response error distribution. Instead, it utilizes the concept of the mean resultant vector of a circular distribution, whose direction reflects the circular mean and whose length is related to the distribution’s concentration, with a uniform distribution having a resultant length of zero. Critically, the resultant for a mixture of distributions is equal to the weighted sum of the resultants for the component distributions (e.g., target and non-target errors). This allows for the estimation of mixture weights without a fitting procedure, simply by calculating the ratios of the individual resultant lengths to the resultant length of the entire response distribution. The resultant vector method was modified from that in Bays (2016a) to take into account the minimum feature separations, with a correction analogous to the one described above for histogram estimates. This method requires more data than parametric methods to achieve a given level of variability, so we estimated swap frequencies based on pooled group-level data instead of participant by participant.

#### 2.2.3 Predicting swap frequencies from cue-feature variability

**Figure 2:**
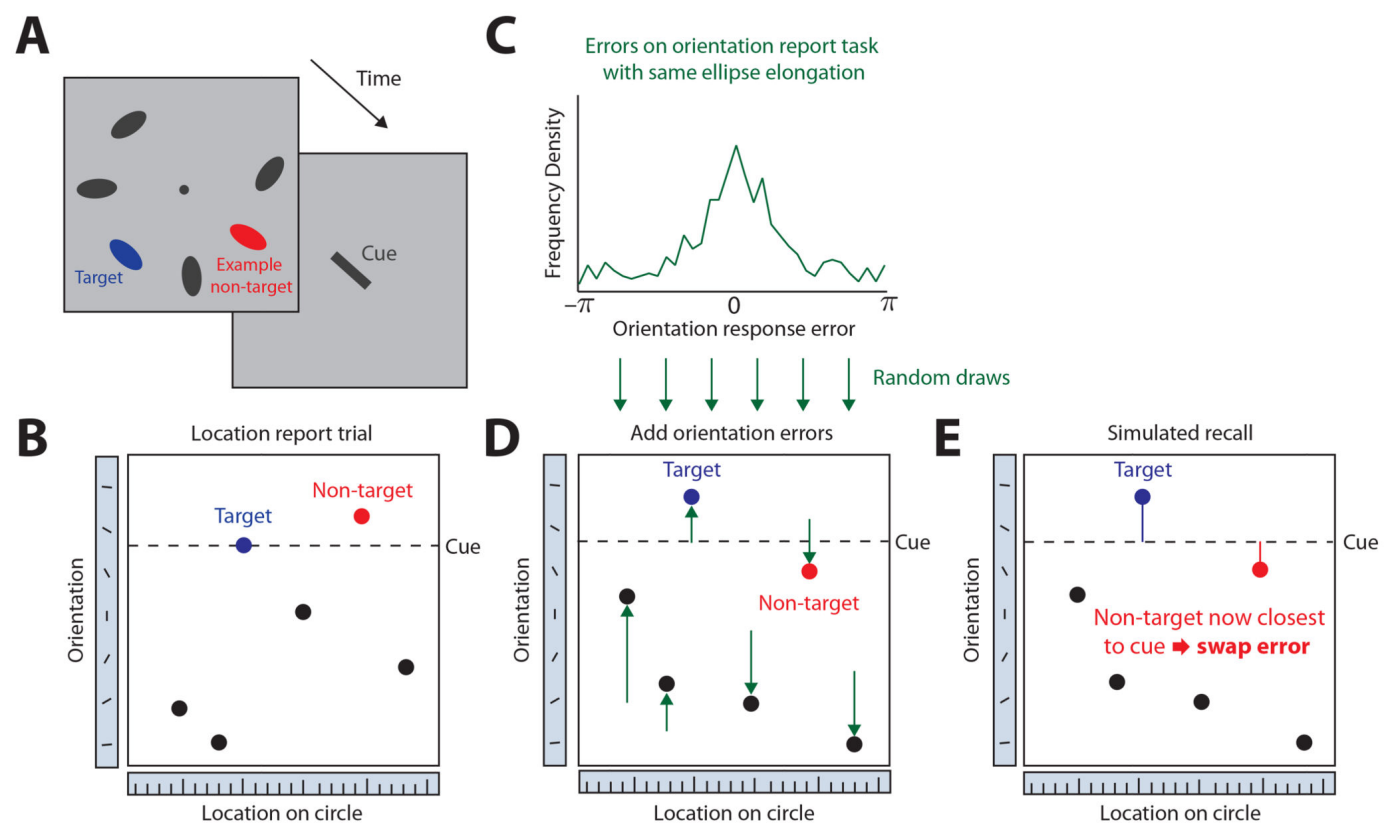
Monte Carlo simulation of swap errors in Experiment 1. **(A & B)** Each trial in the location-report condition (A) is represented by six orientation-location pairs, one for each item, along with the cued orientation (B). **(C & D)** For each such trial, six response errors were drawn at random from trials in the corresponding orientation-report condition (C) and added to the six item orientations on the location-report trial (D). **(E)** If, after the addition of simulated error, the closest orientation to the cued value now belonged to one of the non-targets, the trial was categorised as a swap error. This process was repeated 1000 times for each location-report trial to obtain a predicted mean swap frequency.

To test whether the observed swap frequencies in the location-report condition could be explained as an effect of memory variability for the cue feature (ellipse orientation), we employed a parameter-free Monte Carlo simulation (Fig. 2). This simulation aimed to predict the proportion of swap errors in the location-report condition directly from the observed report errors in the orientation-report condition, and was implemented as follows: For each trial of the location-report task, six orientation error values (deviations from the target orientation) were randomly selected with replacement from the full set of responses made by the same participant with the same ellipse elongation in the orientation-report condition. These errors were added to the orientation feature values of the six items in the location-report trial, simulating variability in memory for these features. Then the circular distance of each resulting orientation from the cue orientation was determined. If the nearest orientation to the cue belonged to one of the non-target items, the simulated trial was considered to have produced a swap error. This process was repeated 1000 times for each trial, separately in each elongation condition, and the mean proportion of swap errors was calculated. We then compared these predicted proportions of swap errors to the values that were estimated from the empirical responses in the location-report condition using the nearest-item heuristic.

#### 2.2.4 Model comparison

Three alternative models of VWM were fit to each participant’s data using the Nelder-Mead simplex method (function fminsearch in Matlab) to produce maximum likelihood estimates of the model parameters (additional model fitting details provided in the Appendix). These were the neural binding model, the interference model and a variant of the interference model with the context-independent activation (A_a_) parameter fixed at zero. Model fit was evaluated using Akaike Information Criterion (AIC) and Bayesian information criterion (BIC) based on each model’s maximum likelihood estimation. Each model is described below, however full details for each model are provided elsewhere (neural binding model: Appendix; interference model: Oberauer & Lin, 2017).

#### 2.2.5 Neural binding model

We fit the neural binding model of Schneegans and Bays (2017) to the data of each participant. This model assumes that the conjunction of features that describes each stimulus (orientation-location in Exp. 1) is encoded in a population of idealized neurons. More specifically, each neuron’s mean firing rate is based on an item’s feature values (cue and report) and the neuron’s preferred value and associated tuning function over the two feature dimensions. Discrete spikes are generated based on this firing rate via a Poisson process. Recall is then modelled as maximum likelihood decoding of the memorized feature values from the noisy neural activity. The item whose decoded cue feature value is closest to the cue in a given trial is selected, and its decoded report feature value is produced as response. This model has three free parameters, namely the tuning curve widths in the two feature dimensions relevant for each task, and the mean total firing rate in the neural population.

We adapted the model described in Schneegans & Bays (2017) to capture the effect of varying the ellipse elongation on memory for orientation. Specifically, we assumed that only a subset of the spikes generated by the neural population carried information about stimulus orientation, while all spikes contributed to the decoding of memorized locations (see also Lugtmeijer et al., 2021). This reflects the fact that all stimuli provided clear and unambiguous location information, but the strength of the orientation signal was reduced. The strength of this effect was captured by an additional *conjunction coding* parameter, that varied between zero (complete absence of orientation information) and one (all spikes contributing equally to the decoding of all features). Depending on whether the affected feature was used as cue or report feature in each task condition, a low value of this parameter would either lead to impaired selection of the cued target item, or to reduced recall precision in reporting the feature of the selected item.

Maximum likelihood fits of the model were obtained for the data of each participant, at each level of ellipse elongation. The model applied six free parameters: the widths of the von Mises tuning curves for orientation, and location, the mean total spike rate in the neural population and the conjunction coding parameter which was allowed to vary as a free parameter between the different elongation levels, but was fixed across the two conditions within each level. All other free parameters were held fixed across all conditions.

In the model, precision is jointly determined by the tuning curve widths and gain parameter. We generated mean precision estimates based on the best fitting model for each participant, with precision expressed as Fisher Information (van den Berg, Shin, Chou, George, & Ma, 2012). Precision estimates were made for all features of the target, or selected non-target, comparing trials where the target item was correctly selected with trials where a swap error occurred. Because estimation is based on a discrete sample of spikes in this model, there is a probability of having zero spikes with which to recover one or both of an item’s features. We assessed the conditional probability across trials that there were no spikes reflecting the features of the target, or selected non-target, again comparing swap and non-swap trials (see Appendix for further details).

#### 2.2.6 Interference model

In order to further evaluate the feature variability account of swap errors, we also fit two variants of the interference model of working memory (Oberauer & Lin, 2017) to the data. This model proposes that representations of feature conjunctions in working memory can be conceptualized as distributions of binding strength in a binding space, spanned by the report-feature dimension and the cue- (or context-) feature dimension. In cued recall, the retrieval cue is fed as an activation pattern into the cue-feature dimension of the binding space. It projects to the report-feature dimension via the pattern of binding strengths reflecting the current memory content, and the response is drawn from the resultant activation pattern over the space of possible report-feature values.

The resulting distribution of response values in this model can be described as a mixture of multiple components. The first component reflects cue-based retrieval, in which each memory item is selected with a probability based on the similarity of its cue-feature to the given retrieval cue (determined via an exponential function of feature distance). The response is then drawn from a von Mises distribution with fixed precision, centered on the chosen item’s report-feature. A second component reflects cue-independent retrieval, in which each item is selected with equal probability, and its report-feature likewise reported with fixed precision. The third component is a uniform distribution, reflecting set-size dependent background noise in the working memory representations. Additionally, the model assumes that in each trial, a single sample item is held in the focus of attention. If this item is the target, it is more likely to be selected in the cue-based retrieval and its report feature value is reproduced with higher precision.

The interference model thus implements two sources of swap errors, one based on cue-feature similarity and one that is cue-independent. To test whether this second source of swap errors is needed, we fit the data both with the full interference model and with a model variant without the cue-independent retrieval. The full interference model has six free parameters, namely the two memory precision parameters for items within and outside of the focus of attention (*κ_f_* and *κ*), the weights of the cue-independent retrieval component (*A_a_*) and of the background noise (*A_b_*; the weight of the cue-based retrieval, *A_c_*, was fixed to one, as in Oberauer and Lin (2017)), the width of the exponential function that determines item selection in the cue-based retrieval (*s),* and finally the proportional reduction of weights *A_a_* and *A_b_* when the target item is in the focus of attention (*r*). For the model variant without cue-independent retrieval, the weight *A_a_* was fixed to zero.

Unlike the neural binding model, the interference model does not make any predictions about the relationship between recall variability in the report of a feature and the frequency of swap errors when that same feature is used as retrieval cue. Therefore, the model was fit to each report feature condition independently.

#### 2.2.7 Toolbox

A MATLAB toolbox implementing the mixture model and resultant vector methods of estimating swaps is available to download from https://bayslab.com/toolbox.

### 2.3 Results

Participants viewed six oriented ellipses and in separate blocks were either cued with the location of one ellipse and had to report its orientation, or they were cued with the orientation of one ellipse and had to report its location. To determine to what degree swap errors are caused by noise in the memory representation of the cue feature, we manipulated the fidelity of the orientation representation by varying the elongation of the ellipse stimuli from trial to trial. Based on the feature-variability account, we expected that this manipulation would affect response variability, but not swap probability, when participants reported stimulus orientation. In contrast, when orientation was used as a cue for reporting stimulus location, we expected to see an effect specifically on the frequency of swap errors.

**Figure 3:**
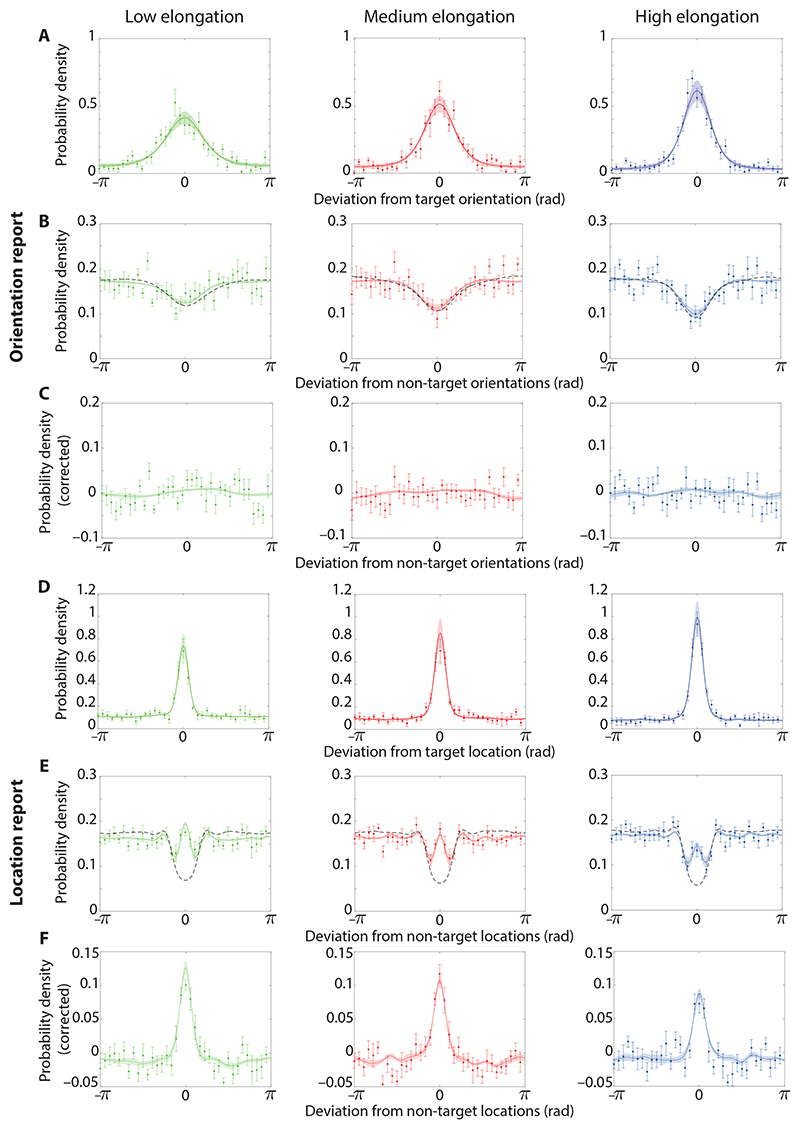
Distributions of responses in Experiment 1. **(A–C)** Orientation recall, based on a location cue. Response distributions are plotted **(A)** relative to the target orientation, **(B)** relative to non-target orientations and **(C)** relative to non-target orientations with the expected distribution in the absence of swap errors (black dashed line in B) subtracted (see Methods), for each elongation condition. **(D–F)** Location recall, based on an orientation cue. Response distributions **(D)** relative to target location, **(E)** relative to non-target locations and **(F)** relative to non-target locations with expected distribution subtracted. In all plots, data points display the behavioural results (error bars indicate ±1 SE) whereas the solid lines indicate the mean results from the fitted neural binding model (shading indicates ±1 SE). Black dashed lines (B and E) indicate the expected distribution in the absence of swap errors applied to the results from the fitted neural binding model. Orientation values are scaled up to the range (−*π* to *π*) to allow easier comparison between features.

Fig. 3A displays the distributions of errors (response deviations from the target) for the three ellipse elongation conditions when recalling orientation. As expected, error variability decreased with increasing elongation (mean circular standard deviation reported as M ± SE, low: 1.18 ± 0.12, medium: 1.07 ± 0.16, high: 0.95 ± 0.19). There was a significant decrease in variability between the medium and high (*t*_9_ = 3.59, p = 0.006, BF_10_ = 9.54) and between the low and high (*t*_9_ = 3.04, p = 0.014, BF_10_ = 4.75) elongation conditions, though the difference between the low and medium conditions was not significant (*t*_9_ = 2.08, p = 0.067, BF_10_ = 1.41). These results indicate that the manipulation of elongation of the ellipses was successful in modulating the variability in the memory representation for orientations.

The distribution of responses relative to the orientations of non-target items are shown in Fig. 3B, for each elongation condition. Fig. 3C displays the same distributions with a correction for minimum feature separations (see Methods). The distribution expected in the absence of swap errors (Fig. 3B, black dashed lines), based on the distribution of non-targets relative to targets, has been subtracted. The absence of a central tendency in these plots indicates that there is little to no evidence for swap errors in the orientation-report condition. To quantify this, we compared the observed MAD between the reported orientation and the non-target orientations to the value expected if non-targets had no influence on responses. There was no significant difference between observed and expected deviation in the medium (1.68 ± 0.03, expected: 1.60 ± 0.03, *t_9_* = 2.20, p = 0.056, BF_10_ = 1.63) and high (1.69 ± 0.03, expected: 1.62 ± 0.02, *t_9_* = 2.15, p = 0.060, BF_10_ = 1.54) elongation conditions. In the low elongation condition the observed deviation was significantly different from the expected one (1.64 ± 0.02, expected: 1.61 ± 0.02, *t*_9_ = 2.89, p = 0.018, BF_10_ = 3.92), but the difference was small and in the opposite direction to that which would be produced by swap errors.

Fig. 3D displays the distributions of errors in the location-report condition for each of the three ellipse elongations. Overall error variability as assessed by circular standard deviation was again influenced by elongation (low: 1.59 ± 0.09, medium: 1.46 ± 0.15, high: 1.19 ± 0.09), with significant differences between medium and high (*t*_9_ = 3.33, p = 0.009, BF_10_ = 6.84), low and high (*t*_9_ = 7.57, p < 0.001, BF_10_ = 715.98) but not low and medium conditions (*t*_9_ = 1.44, p = 0.183, BF_10_ = 0.69). In this case, however, we found evidence that these changes reflected differences in the frequency of reporting non-targets rather than variability in memory for location itself.

The response distributions centered on the locations of non-target items, corrected for the effects of minimum separation, are shown in Fig. 3F. They display a clear central tendency suggesting the presence of swap errors when reporting location. Supporting this, the MAD between the reported location and the non-target locations was significantly lower than the value expected in the absence of swap errors for every elongation condition (low: 1.59 ± 0.02, expected: 1.68 ± 0.01, *t*_9_ = 5.23, p < 0.001, BF_10_ = 68.54; medium: 1.60 ± 0.02, expected: 1.71 ± 0.01, *t*_9_ = 7.43, p < 0.001, BF_10_ = 627.91; high: 1.64 ± 0.02, expected: 1.70 ± 0.01, *t*_9_ = 3.64, p = 0.005, BF_10_ = 10.24).

#### 2.3.1 Comparison with simulated swap frequencies

**Figure 4:**
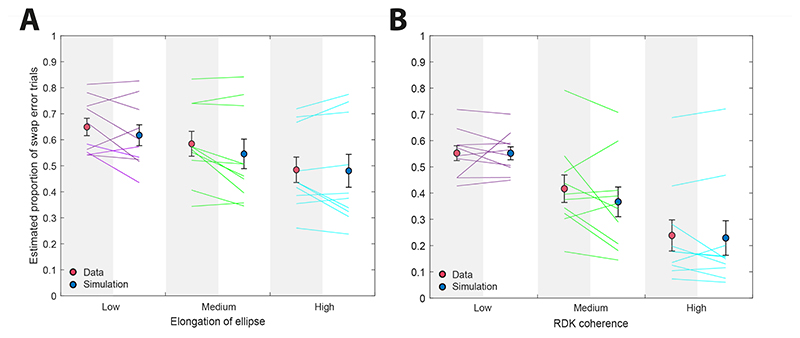
Swap error estimates. **(A)** Experiment 1. The proportion of trials in which a swap error occurred estimated from the data (red) or the simulation (blue) across the three different levels of ellipse elongation. **(B)** Experiment 2. The proportion of trials in which a swap error occurred estimated from the data (red) or the simulation (blue) across the three different levels of RDK coherence. Coloured lines show estimates for individual participants. The error bars indicate the ±1 SE.

Qualitatively, the pattern of results in Figs. 3D, E & F suggests that the manipulation of orientation variability affected the frequency of swap errors when orientation was the feature used to cue which item to report. To quantify this effect, we calculated a non-parametric estimate of swap error frequency in the location-report condition based on a nearest-item heuristic (see Methods). This estimate ranged from 48% ± 5% of trials with high elongation to 65% ± 3% with low elongation, with the estimated frequency increasing as ellipse elongation decreased (Fig. 4A). There was a significant increase in swap error frequency between the low and medium (*t*_9_ = 2.49, p = 0.034, BF_10_ = 2.35), medium and high (*t*_9_ = 4.46, p = 0.002, BF_10_ = 28.08), and low and high (*t*_9_ = 5.24, p < 0.001, BF_10_ = 68.66) elongation conditions.

The key question we aim to answer in the present study is whether the observed changes in the frequency of swap errors when reporting location can be explained in full by differences in memory variability for orientation. To address this question, we employed a Monte Carlo simulation that predicts swap frequency in the location-report condition from response errors in the orientation-report condition. This simulation is based on three assumptions: first, that the response errors in the orientation-report condition for a given ellipse elongation accurately reflect memory variability for orientation; second, that this memory variability will be the same independent of whether orientation is used as cue or report feature; and third, that to make a response, participants will compare the given cue with the (imprecise) cue feature values of all items retrieved from memory, choose the item that is closest to the given cue, and report its retrieved response feature value (compare Schneegans & Bays, 2017). Note that the first assumption would be violated if a considerable amount of swap errors occurred in the report-orientation condition (because then the response errors in this condition would not reflect purely memory variability for orientation), but our analysis of orientation report errors showed that this was not the case.

Based on these assumptions, we simulated the occurrence of swap errors in the location-report condition as illustrated in Fig. 2. We added observed recall errors from the orientation report condition to the actual orientation features of each location-report trial, separately for each participant and elongation condition, and determined how often this would lead to a non-target item being selected as the most likely target (because its retrieved orientation was closest to the given cue; see Methods for details). As shown in Fig. 4A, the frequency of swap errors predicted by the simulation (blue) closely approximated the swap frequencies observed in the data (red) at each elongation level, with no significant difference in the low (*t*_9_ = 1.32, p = 0.22, BF_10_ = 0.61) and the high (*t*_9_ = 0.63, p = 0.55, BF_10_ = 0.37) elongation conditions (Fig. 4A). There was a borderline significant difference in the medium elongation condition according to the frequentist *t*-test, but the Bayes factor indicated only very weak evidence for a difference (*t*_9_ = 2.3, p = 0.047, BF_10_ = 1.85).

#### 2.3.2 Validating swap estimates

To facilitate comparison with the simulation, the empirical swap estimates above were based on a simple nearest-item heuristic. To confirm their validity we compared these estimates to those produced using two established methods: the three-component mixture model (Bays et al., 2009) and the resultant vector method outlined in Bays (2016a).

Based on fitting the mixture model, the mean probability of swap errors (non-target responses) in the orientation-report condition was low and did not change significantly with ellipse elongation (low elongation = 15% ± 5%, medium elongation = 16% ± 9%, high elongation = 6% ± 4%). Conversely, the estimated probability of swap errors in the location-report condition was overall quite high, and decreased with increasing elongation (low = 57% ± 4%, medium = 53% ± 5%, high = 39% ± 5%), with significant differences between the low and high (*t*_9_ = 2.72, p = 0.024, BF_10_ = 3.14) and medium and high conditions (*t*_9_ = 5.89, p < 0.001, BF_10_ = 139.95), though the difference between the low and medium conditions was not significant (*t*_9_ = 0.57, p = 0.583, BF_10_ = 0.35).

Based on the resultant vector method, which was applied to pooled data, in the orientation-report condition the swap error estimates decreased from 39% in the low elongation condition to 14% and 10% in the medium and high elongation conditions respectively. Similarly, in the location-report condition a high proportion of swap errors was estimated in the low elongation condition (57%) which decreased to 54% and 36% in the medium and high elongation conditions.

Overall, the estimates obtained with both methods for the location-report condition follow a similar pattern to those based on the nearest-item heuristic, in that we found an increase in swap errors as ellipse elongation decreased. Furthermore, swap frequencies from both methods for the orientation-report condition were mostly low which is congruent with the distributions of response errors in Fig. 3C. The one notable disagreement is in the low elongation condition where the resultant vector swap estimate was higher than the mixture model estimate. Estimates of swap frequency become more variable as report-dimension variability increases, because of the increasing overlap of response probability distributions for swap and non-swap responses. This may explain the discrepancy occurring in the low elongation condition where orientation report variability was greatest.

#### 2.3.3 Model comparison

To further explore the mechanisms underlying swap errors, the behavioural data from each participant were fit with three parametric models of VWM. This included the neural binding model that represents feature binding through conjunctive coding. Recall is captured as decoding of memorized feature values from noisy neural activity. Specifically, the item whose decoded cue feature value is most similar to the cue is selected and its decoded report feature value is output as the response.

Swap errors in the model occur when, due to decoding errors, the cue feature of a non-target item is deemed more similar to the cue than the target (which exactly matches the cue). In order to capture the effect of varying ellipse elongation on orientation estimates, we included a free parameter for each level of elongation that changed how much information about orientation was encoded in the spiking activity, while leaving the information about location unchanged (see Methods for details).

Each participant’s behavioural data was also fit with full and partial versions of the interference model. Within the full model, recall involves using the context feature to retrieve a bound report (content) feature. Use of the context feature as a cue leads to re-activation of target and non-target features and the most highly activated feature is likely to be retrieved. Swap errors in this model can be caused either by context-dependent activation which closely follows the feature variability account or by context-independent activation which is not predicted by the feature variability account. The partial interference model fixes context-independent activation at zero to investigate the ability of cue-dependent processes to fully account for swap errors in the current task.

Both the neural binding model and interference model successfully reproduced the distributions of response errors across all elongations in both orientation and location report conditions. However, quantitative comparisons using AIC and BIC demonstrated that the neural binding model consistently provided a better fit to the data (Table. 1). Furthermore, the fit of the partial interference model improved upon the full model by on average 10.61 and 35.02 per participant for AIC and BIC respectively.

Summaries of the estimated parameters are provided for the neural binding model and both versions of the interference model in the Appendix. Of note is the parameter representing context-independent activation (A_a_) which remains close to zero across conditions.

As the best fitting model, the predictions of the neural binding model are explored in more detail below. Predictions of the model, with best fitting parameters for each participant are also shown by solid lines in Fig. 3.

#### 2.3.4 Neural binding model

The neural model does not implement any explicit guessing strategy that would produce swap errors specifically when the cued item is not held in memory, nor does it implement an upper limit on the number of items or features stored. However, due to the assumed stochasticity of neural activity, it is possible that no spikes contribute to the decoding of one or both of the individual feature values, in which case the decoded feature value is drawn from a uniform distribution. We assessed how these zero-spike cases contributed to the occurrence of swap errors in the model. In consideration of our main hypothesis, we focus here on the representation of the cue feature in the location-report condition, the condition where we predominantly observe swap errors. Results for the other conditions are reported in the Appendix.

Based on the neural model with best fitting parameters for each participant, group median conditional frequency with which the target received zero spikes in the cue dimension given that a swap error occurred was 31% (IQR: 11%-56%) in the low elongation condition, 13% (IQR: 5%-48%) in the medium elongation and 3% (IQR: 0%-29%) in the high elongation condition. Using the same method, we also determined the median probability on swap trials that the reported non-target item received zero spikes in the cue dimension: this was comparable to the results for the target item (low: 26%, IQR = 10%-51%; medium: 11%, IQR = 4%-44%; high: 2%, IQR = 0%-24%). In comparison, the median conditional probability that the target item received zero spikes in the cue dimension if the target item was correctly selected remained low across conditions, decreasing as elongation increased (low: 8%, IQR = 4%-27%; medium: 3%, IQR = 1%-21%; high: 0%, IQR = 0%-9%). All estimated probabilities were significantly different within levels of elongation as determined using a Wilcoxon signed-rank test (all p < 0.01, BF_10_ > 23.32).

These results suggest that a majority of swap errors occurred in cases where information about the target’s cue feature was available. To examine whether there was a relationship with the quality of this representation, we used the fitted neural model to calculate the mean precision (expressed in terms of Fisher Information) for items in swap and non-swap trials. This analysis showed that the mean cue-feature precision for target items was higher on trials where the target item was correctly selected for report (low: 1.59 ± 0.33, medium: 2.23 ± 0.45, high: 3.21 ± 0.60) than on swap trials (low: 1.09 ± 0.22, medium: 1.69 ± 0.35, high: 2.61 ± 0.49). On swap trials, mean precision for selected non-target items (low: 1.20 ± 0.25, medium: 1.82 ± 0.38, high: 2.78 ± 0.53) was marginally higher than for target items. The mean precision estimates were all significantly different within levels of elongation (all p < 0.03, BF_10_ > 2.57).

### 2.4 Discussion

In Experiment 1, the elongation of ellipse stimuli was varied with the purpose of manipulating memory variability for their orientations. The results of the orientation report condition indicated that this manipulation was successful, as demonstrated by increases in response variability with decreasing ellipse elongation. In the location report condition, where orientation was used as the cue feature, we observed an increased concentration of responses around non-target locations as ellipse elongation decreased.

This pattern of results is consistent with the feature-variability account of swap errors, whereby increased variability in recall of item orientations, when orientation is used to indicate which item to report, results in an increasing probability of erroneously reporting a non-target item. In order to determine whether swap errors could be explained in their entirety by this mechanism, we simulated responses in the location report condition based on the errors observed in the orientation report condition, using a nearest-item heuristic to estimate swap frequency. The predicted swap frequencies very closely matched the observed frequencies (Fig. 4) including the changes induced by varying ellipse elongation.

A model comparison of parametric models favoured the neural binding model over full and partial versions of the interference model. The cause of swap errors in the neural binding model closely follows the feature variability account, whereas the interference model includes an additional cue-independent source of swap errors. In the full model, the estimated parameter representing cue-independent activation remained close to zero across conditions. Moreover, the partial model with this parameter fixed at zero was a better fit than the full model. Overall, this provides further support for the hypothesis that swap errors can be fully accounted for by variability in memory for the cue feature.

Additional analysis was carried out based on the neural binding model to explore how often swap errors occurred when zero spikes contributed to the decoding of one or both of the feature values. Results from the location-report condition indicated that the majority of swap errors occurred in cases where information about the target’s cue feature was available. Furthermore, swap errors were more likely to occur when memory precision for both the target and non-target’s cue feature was low suggesting that these responses may be associated with low confidence ratings. This is explored further in the General Discussion. Overall, the results of Experiment 1 support the hypothesis that variability in the cue-feature dimension is sufficient to fully explain swap errors in cued recall.

## 3 Experiment 2

In order to determine whether the effect of orientation cue variability on swap errors could be replicated in another feature dimension, in Experiment 2 motion direction took the place of orientation. Participants were shown four motion stimuli at different locations and asked to report one item’s direction of motion or location after being cued with the alternate feature. The variability of the motion direction representation was manipulated via motion coherence, which was varied across trials.

### 3.1 Methods

#### 3.1.1 Experimental protocol

Ten participants, reporting normal or corrected-to-normal visual acuity, took part in the second experiment (aged 18–29 years; three male, seven female). The task (illustrated in Fig. 1B) was identical to Experiment 1 with the following exceptions. Instead of ellipses, each memory array consisted of four motion stimuli (random dot kinematograms, RDK). Each RDK consisted of 45 dark grey dots (diameter, 0.1° of visual angle) moving at a constant speed of 5°/s within a circular aperture (diameter, 2° of visual angle) bounded by an annulus of the same colour. Dot lifetime was unlimited and dots reaching the boundary of the circle were repositioned at the same point on the opposite side, maintaining a constant dot density. Instead of ellipse elongation, RDKs varied from trial to trial between three levels of motion coherence: 30%, 60% or 100% (low, medium or high coherence) defined as the proportion of dots moving in the same direction (dots that were not coherent were assigned random directions). The coherent motion direction for each RDK was chosen at random, with a minimum separation of 60° between the directions of different RDKs within a trial. The locations of the stimuli were also chosen at random with a minimum separation between the centers of each RDK of 60° on the circle, matching the motion separation. Locations were cued and reported in the same way as in Experiment 1. Motion directions were cued and reported with a centrally presented dark grey arrow (length 3°, width 0.1°).

### 3.2 Analysis

The analysis for Experiment 2 was equivalent to the analysis conducted for Experiment 1, with motion direction and motion coherence in place of orientation and ellipse elongation.

### 3.3 Results

**Figure 5:**
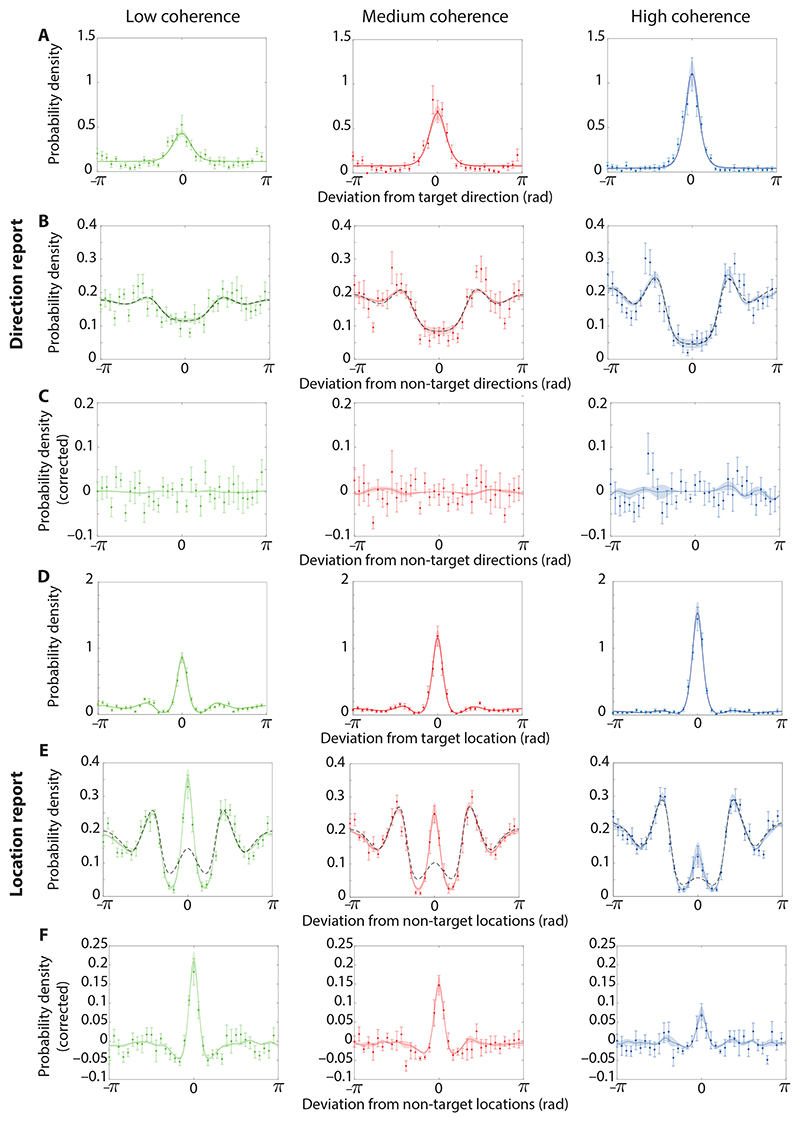
Distributions of responses in Experiment 2. **(A–C)** Direction recall, based on a location cue. Response distributions are plotted **(A)** relative to the target direction, **(B)** relative to non-target directions and **(C)** relative to non-target directions with expected distribution in the absence of swap errors (black dashed line) subtracted (see Methods), for each coherence condition. **(D–F)** Location recall, based on a direction cue. Response distributions **(D)** relative to target location, **(E)** relative to non-target locations and **(F)** relative to non-target locations with expected distribution subtracted. In all plots, data points display the behavioural results (error bars indicate ±1 SE) whereas the solid lines indicate the mean results from the fitted neural binding model (shading indicates ±1 SE). Black dashed lines (B and E) indicate the expected distribution in the absence of swap errors applied to the results from the fitted neural binding model.

Fig. 5A displays the error distributions when reporting direction across the three RDK coherence conditions in Experiment 2. There was a significant decrease in variability (mean circular standard deviation, low: 1.63 ± 0.05, medium: 1.22 ± 0.18, high: 0.80 ± 0.16) between the low and medium (*t*_9_ = 2.57, p = 0.03, BF_10_ = 2.58), medium and high (*t*_9_ = 8.46, p < 0.001, BF_10_ = 1540.56), and low and high (*t*_9_ = 5.54, p < 0.001, BF_10_ = 96.29) coherence conditions.

The error distributions centred on the motion directions of non-target items are shown in Fig. 5B). The undulating patterns, with peaks in the response distribution at approximately ±*π*/2 (±90°) and *±π* (±180°) relative to the non-target, are a consequence of the relatively large minimum distance (60°) enforced between the stimulus directions on a trial, which in combination with the set size of four resulted in a higher probability of target and non-target directions differing by these angles. Confirming this interpretation, the same pattern was present in the distribution expected in the absence of swap errors (dashed black line in Fig. 5B). Subtracting the expected distribution left an approximately uniform function without central tendency (Fig. 5C), indicating few to no swap errors occurred when reporting motion direction. The MAD between the reported motion direction and the non-target directions was not significantly different from the value expected in the absence of swap errors for any coherence condition (low: 1.68 ± 0.01, expected: 1.67 ± 0.02, *t*_9_ = 0.37, p = 0.72, BF_10_ = 0.33; medium: 1.77 ± 0.04, expected: 1.77 ± 0.03, *t*_9_ = 0.27, p = 0.79, BF_10_ = 0.32; high: 1.84 ± 0.04, expected: 1.87 ± 0.03, *t*_9_ = 1.64, p = 0.14, BF_10_ = 0.85).

Fig. 5D displays the error distribution when reporting location across the three RDK coherence conditions. There was a significant decrease in overall variability (mean circular standard deviation, low: 1.60 ± 0.10, medium: 1.25 ± 0.15, high: 0.81 ± 0.16) between the low and medium (*t*_9_ = 4.05, p = 0.003, BF_10_ = 17.03), medium and high (*t*_9_ = 7.36, p < 0.001, BF_10_ = 590.84), and low and high (*t*_9_ = 9.02, p < 0.001, BF_10_ = 2416.41) coherence conditions. Like the equivalent changes observed in Experiment 1, we found evidence that this result is due to changes in swap frequency rather than an actual difference in variability of memory for location information across conditions.

The error distributions centred on the locations of non-target items all displayed clear central tendencies after correcting for the effects of minimum feature separation (Fig. 5F), suggesting the presence of swap errors when reporting location. The MAD between the reported location and the non-target locations was significantly different from the expected value in the low (1.63 ± 0.02, expected: 1.71 ± 0.02, *t*9 = 6.10, p < 0.001, BF_10_ = 173.93), medium (1.74 ± 0.03, expected: 1.78 ± 0.03, *t*9 = 3.79, p = 0.004, BF_10_ = 12.37) and high coherence conditions (1.84 ± 0.04, expected: 1.87 ± 0.03, *t*9 = 3.29, p = 0.009, BF_10_ = 6.52).

#### 3.3.1 Comparison with simulated swap frequencies

We expected that manipulating coherence would elicit greater changes in variability than produced by ellipse elongation in Experiment 1, resulting in even stronger effects on swap error frequency, and this was indeed the case. Swap error frequency in location report, as estimated by the nearest-item heuristic, decreased strongly with increasing motion coherence (low: 55% ± 3%, medium: 42% ± 5%, high: 24% ± 6%; all p < 0.005, BF_10_ > 12.6; Fig. 4B).

As in Experiment 1, we used a Monte Carlo simulation based on the cue-feature variability account to predict the swap frequencies in the location-report condition from response errors in the direction-report condition. Consistent with the estimates obtained from the nearest-item heuristic, predicted swap frequencies decreased significantly as motion coherence increased (low: 55% ± 3%, medium: 36% ± 6%, high: 22% ± 7%; all p < 0.002, BF_10_ > 28.3). Critically, the predicted swap frequencies closely matched the estimated values, showing no significant difference between estimate and prediction at any coherence level (all p > 0.15, BF_10_ < 0.77).

#### 3.3.2 Validating swap estimates

The mean probability of swap errors (non-target responses) estimated using the mixture model (Bays et al., 2009) in the direction-report condition was low and did not change significantly with variation in the motion coherence (low coherence = 4% ± 2%, medium coherence = 6% ± 2%, high coherence = 12% ± 7%). Conversely, the estimated probability of swap errors in the location-report condition increased with decreases in coherence with significant differences between the low and high (low = 51% ± 2%, high = 19% ± 6%, *t*_9_ = 5.25, p < 0.001, BF_10_ = 70.01), medium and high (medium = 38% ± 5%, *t*_9_ = 5.98, p < 0.001, BF_10_ = 152.99) and low and medium conditions (*t*_9_ = 2.98, p = 0.016, BF_10_ = 4.36).

In the direction-report condition, the swap error estimates produced from the pooled group data using the resultant vector method (Bays, 2016a) remained predominantly low ranging from −5% in the low coherence condition to 2% and 21% in the medium and high coherence conditions respectively (note that a negative estimate of swap frequency obtained by this method can be interpreted as strongly favouring no swaps in the data). In contrast, in the location-report condition a high proportion of swap errors was estimated in the low coherence condition (55%) which decreased to 32% and 26% in the medium and high coherence conditions, respectively.

Overall, similarly to Experiment 1, all estimation methods consistently indicated that swap errors in the location-report condition increased as motion coherence decreased. Additionally, when reporting direction the estimated swap errors remained low with no evident link to the motion coherence condition which is congruent with the error distributions in Fig. 5C.

#### 3.3.3 Model comparison

The behavioural data from each participant were again fit with three models of VWM. This included the neural binding model as well as the full and partial interference models.

Both the neural binding model and interference model successfully reproduced the distributions of response errors across all levels of coherence in both direction and location report conditions. However, quantitative comparisons using AIC and BIC indicated that the neural binding model consistently provided a better fit to the data for all but one participant whose data was fit better by the full interference model based on AIC values only (Table. 2). The comparison of full and partial interference models produced mixed findings with AIC indicating that the full model was a better fit, while BIC, which more heavily penalizes free parameters, favoured the partial model.

Summaries of the estimated parameters are provided for the neural binding model and both versions of the interference model in the Appendix. In contrast to the results of Experiment 1, the parameter representing context-independent activation (A_a_) deviated substantially from zero in some levels of coherence in the location-report condition.

The predictions of the neural binding model are explored in more detail below. Predictions of the model, with best fitting parameters for each participant are also shown by solid lines in Fig. 5.

#### 3.3.4 Neural binding model

The neural binding model was again used to explore how often swap errors occurred in the location-report condition when the target item was attributed zero samples in the cue dimension (motion direction). The group median conditional probability that the target received zero samples in the cue dimension if a swap error occurred was 89% (IQR: 88%-91%) in the low coherence condition, 83% (IQR: 80%-88%) in the medium coherence and 75% (IQR: 67%-76%) in the high coherence condition.

The group median conditional probability that the selected non-target item received zero samples in the cue dimension if a swap error occurred was consistently lower than the result for the target item (low: 82%, IQR = 79%-83%; medium: 70%, IQR = 60%-77%; high: 39%, IQR = 36%-51%). Furthermore, the group median conditional probability that the target item received zero samples in the cue dimension if the target item was correctly selected was lower still across conditions (low: 44%, IQR = 43%-51%; medium: 28%, IQR = 18%-34%; high: 7%, IQR = 5%-12%), though substantially higher than the probabilities observed in Experiment 1. Furthermore, all estimated probabilities were again significantly different within levels of coherence as determined using a Wilcoxon signed-rank test (all p < 0.002, BF_10_ > 31.82).

The group mean cue-feature precision was again higher for target items in trials where the target item was correctly selected for retrieval (low: 4.02 ± 0.64, medium: 6.67 ± 0.99, high: 11.96 ± 2.01) than both target items (low: 0.70 ± 0.11, medium: 1.40 ± 0.23, high: 2.72 ± 0.42) and selected non-target items (low: 1.24 ± 0.22, medium: 2.90 ± 0.56, high: 7.37 ± 1.49) in swap error trials. Furthermore, the mean precision estimates were again all significantly different within levels of coherence (all p < 0.003, BF_10_ > 20.67).

### 3.4 Discussion

In Experiment 2, we aimed to determine whether the effect of orientation cue variability on swap errors could be replicated with a different feature dimension. Instead of orientation, this experiment manipulated the memory variability for motion direction through changes in motion coherence. The results closely replicated those of Experiment 1, including increases in response error when reporting direction with decreasing motion coherence, and an increased concentration of responses around non-target locations when cueing with motion direction.

Furthermore, predicted swap frequencies based on simulated location responses once again closely matched the observed frequencies across levels of motion coherence. This was despite the range of swap error frequencies covered by the manipulation of coherence being substantially larger than in Experiment 1.

The model comparison replicated the result of Experiment 1 favouring the neural binding model over full and partial versions of the interference model. However, the comparison of the full and partial interference model was less consistent than Experiment 1 and the best fitting model changed dependent on the criterion used. Furthermore, the cue-independent activation parameter (A_a_) was substantially above zero in some levels of coherence in the location-report condition. One difference between the two experiments is the minimum feature separation which was greater in this experiment (60°) than Experiment 1 (30°). The increased distance between each item’s cue-feature may increase the likelihood that swap errors are attributed to cue-independent processes. However, the neural binding model remained the best fitting model overall, consistent with the hypothesis that swap errors can be fully accounted for by variability in memory for the cue feature.

Unlike Experiment 1, analysis using the neural binding model showed that in the location-report condition a substantial proportion of swap errors occurred in cases where information about the target’s cue feature was not available. The model accounted for errors on these trials according to the same mechanism as trials where target information was available. Furthermore, swap errors were again more likely to occur when memory precision for both the target and non-target’s cue feature was low suggesting that these responses may be associated with low confidence ratings. Overall, the results of Experiment 2 provide further support for the proposal that variability in memory for the cue feature can fully explain swap errors.

## 4 Experiment 3

The results of Exps. 1 and 2 indicated that swap error frequencies on the location-report tasks were fully accounted for by variability in the cue feature, as estimated in a separate task. This suggests that cue-independent swaps, of the kind predicted by failures of feature binding independent of individual features, did not occur in these tasks. Superficially, this finding seems in conflict with a previous study by Oberauer and Lin (2017) which found evidence in fitted model parameters for cue-independent as well as cue-dependent swaps. One difference between this and the previous study is that we chose stimulus features, including location, randomly from a uniform distribution on the circle, whereas stimulus locations in the previous study were chosen from a fixed set of evenly-spaced points on the circle that stayed the same from trial to trial. Such predictability in spatial configuration has also been raised by other authors as a possible influence on swap errors (Brady & Störmer, 2020; Schurgin et al., 2020).

**Figure 6:**
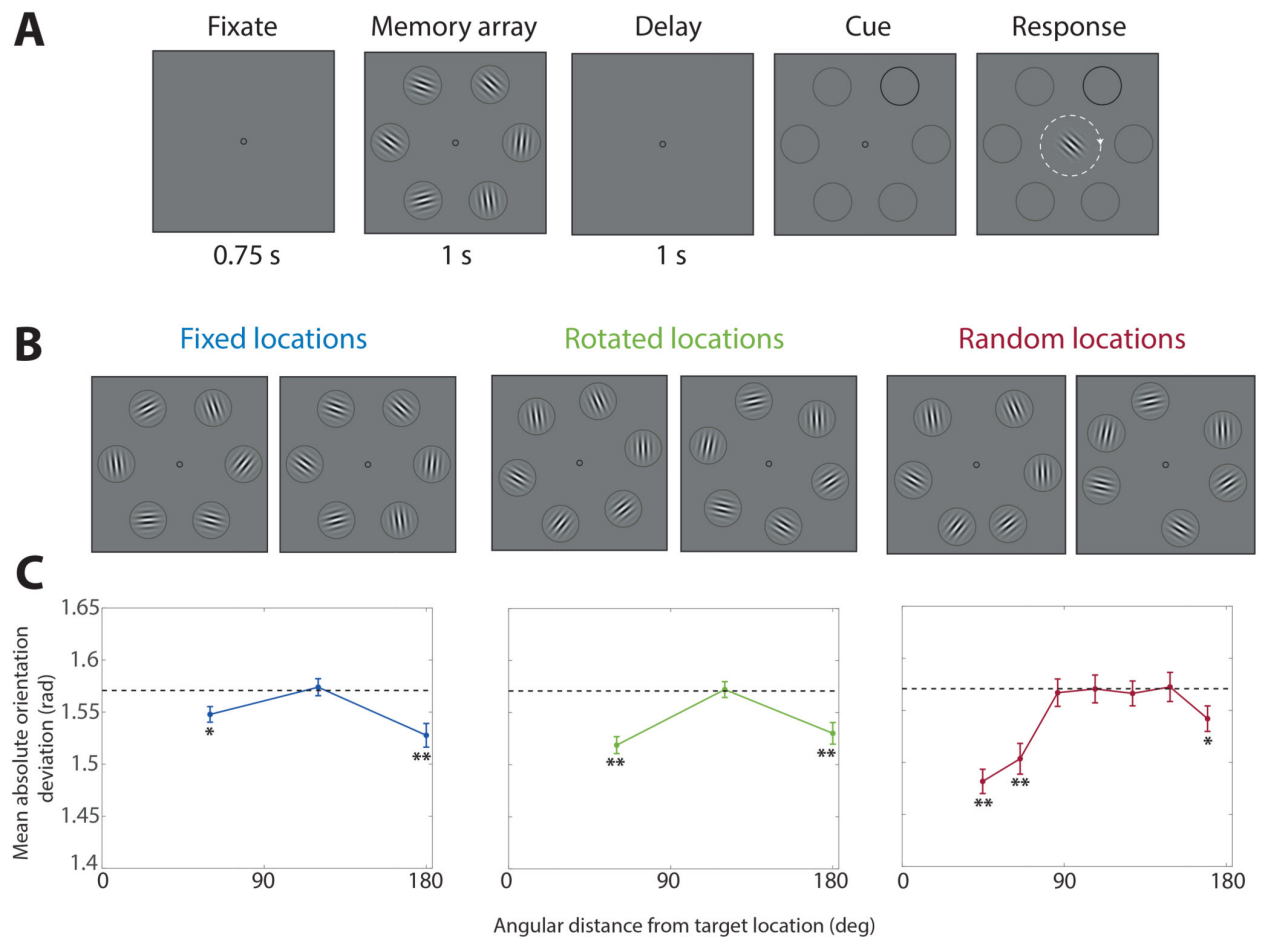
Experiment 3. **(A)** Experiment design. A stimulus array was presented on each trial consisting of six Gabor patches. After a delay, participants were cued with the location of one patch from the preceding array, and used their cursor to report the orientation. Displays are not to scale. **(B)** Memory array examples. The locations of the Gabor patches were either fixed for all trials and evenly spaced **(Left)**, evenly spaced but rotated each trial **(Middle)** or randomly generated each trial with a minimum separation **(Right)**. **(C)** The MAD of orientation responses from each non-target orientation as a function of spatial (angular) distance between the target and the non-target. **(Left)** For the fixed location condition; **(Middle)** for the rotated location condition. **(Right)** for the random location condition. Orientation values are scaled up to the range (−π to π). Error bars indicate ±1 SE. Asterisks indicate significant differences from expected level (marked by dashed line): * p < 0.05, ** p < 0.001.

To test this, Experiment 3 comprised an orientation report task with a spatial cue (Fig 6A) and three between-participant experimental conditions (illustrated in Fig 6B). Participants were shown memory arrays of six oriented Gabor patches, where the locations of the six Gabors were either evenly distributed around an invisible circle and fixed for all trials, evenly distributed but with a different random rotation of the whole array on each trial, or randomly distributed on every trial.

### 4.1 Methods

#### 4.1.1 Experimental protocol

254 online participants carried out a cued recall task testing their memory for orientation and location. All participants were recruited using Prolific (https://www.prolific.co), reported normal or corrected-to-normal visual acuity and gave informed consent in accordance with the Declaration of Helsinki. Eleven participants were excluded from the analysis for one of the following reasons: they completed a high number of trials without adjusting the orientation to make their response (two participants); they did not complete all trials (six participants); they performed at chance level in both experimental and catch trials (two participants); inattention was indicated by the extended duration of their testing session (one participant). 243 participants (aged 18–36 years; 106 male, 129 female, 1 transgender, 7 not specified) were therefore included in the analysis.

Each trial of the task (illustrated in Fig. 6A) began with the presentation of a dark grey central fixation circle (diameter, 10 pixels; online experiment stimuli are described in pixels rather than degrees of visual angle due to varying displays) against a mid-grey background. Participants were instructed to keep looking at the fixation circle throughout each trial. After 0.75 s, a memory array consisting of six Gabor patches (width and height, 126 pixels; wavelength of sinusoid, 38 pixels/cycle; s.d. of Gaussian envelope, 20 pixels) was presented for 1 s, outlined by light grey circles which served as placeholders for the patches. The center of each Gabor was positioned on an invisible circle with a radius of 250 pixels centered on the fixation circle. The Gabors’ orientations were chosen at random (without minimum separations). Participants were randomly assigned into one of three conditions (see Fig. 6B). These were the fixed, rotated and random location conditions. In the fixed location condition, the six Gabor patches were presented in the same six locations on every trial, equally distributed around the circle (arc length of 60° between patch centers). In the rotated location condition, the Gabor patches were again equally distributed around the circle but the whole array was randomly rotated on each trial. In the random location condition, each Gabor’s location on each trial was randomly chosen from a uniform distribution on the circle, with a minimum separation (arc length) of 36° between patch centers.

After the presentation of the memory array, the fixation circle was shown for a further 1 s followed by the cue display. The circle outlines reappeared in the locations of the previously presented Gabor patches. One of the outlines was a darker grey to cue the location of the randomly selected target item for that trial. Participants were instructed to begin moving their cursor once they were ready to respond with the orientation of the target Gabor. Once participants moved their cursor, a Gabor patch was presented centrally and participants freely rotated it using their cursor until it matched the remembered orientation of the target. Each participant completed approximately 109 trials. The trials were split into two blocks separated by a one minute minimum break, with the complete testing session lasting approximately 15 minutes.

### 4.2 Analysis

As in previous experiments, orientation values were scaled up to cover the same range as location (−*π* to *π*) for easier comparison of results across features. To examine the pattern of swap errors in each condition, the MAD of reported orientations from non-target orientations was calculated and plotted as a function of the non-target’s angular distance from the cued location. Deviations were compared with the value expected in the absence of swap errors (*π*/2) using Bayesian (using JASP, 2019) and frequentist one-sample t-tests. Since the orientation of each item was generated at random, it was not necessary to account for minimum separations when calculating the expected MAD (as was done for Experiments 1 and 2). MADs significantly below the expected level indicate biases towards non-target orientation values indicative of swap errors.

Similarly to Experiments 1 and 2, swap error frequencies were estimated for each condition using the mixture model method (Bays et al., 2009) applied to subject-level data and the resultant vector method of Bays (2016a) applied to pooled group-level data.

### 4.3 Results

To assess effects of cue similarity, Fig. 6C displays the MAD of reported orientations from non-target orientations, plotted as a function of the non-target’s angular distance from the cued location. Deviations below the value expected in the absence of swap errors (*π*/2; dashed line) in these plots indicate biases towards non-target orientation values indicative of swap errors.

For the fixed-location and rotated-location conditions, non-targets were located at one of three possible angular distances from the cue: 60°, 120° and 180°. In both conditions, the pattern of MADs (Fig. 6C, left and middle) suggested an approximately equal probability of swaps to the 60° (nearest) and 180° (diametrically opposite) non-target orientations, with no evidence for swaps to the intermediate non-targets at 120° from the cue.

The MAD in the fixed-location condition was significantly lower than expected for the non-targets at 60° from the target (MAD = 1.55 ± 0.01, *t*_80_ = 3.10, p = 0.003, BF_10_ = 10.02) and for non-targets positioned precisely opposite to the target (180°; MAD = 1.53 ± 0.01, *t*_80_ = 3.81, p < 0.001, BF_10_ = 77.85). The MAD for the non-target items two positions away (120°) was not significantly different from the expected value (MAD = 1.57 ± 0.01, *t*80 = 0.36, p = 0.723, BF_10_ = 0.13).

In the rotated location condition (Fig. 6C middle), the MAD was once again significantly lower than expected for non-targets at 60° (MAD = 1.52 ± 0.01, *t*_80_ = 6.42, p < 0.001, BF_10_ = 1,274,140) and 180° (MAD = 1.53 ± 0.01, *t*_80_ = 3.93, p < 0.001, BF_10_ = 115.90), but not 120° (MAD = 1.57 ± 0.01, *t*_80_ = 0.15, p = 0.882, BF_10_ = 0.12).

In the random location condition (Fig. 6C right), for purposes of analysis we discretized the distance between non-targets and cue into seven bins of equal interval. The MAD for non-target items in the two bins closest to the cued location (Distance bin 1 = 34°-55°, MAD = 1.48 ± 0.01, *t*_80_ = 7.65, p < 0.001, BF_10_ = 238,478,000; Distance bin 2 = 55°-76°, MAD = 1.50 ± 0.01, *t*_80_ = 4.57, p < 0.001, BF_10_ = 1009.32) and the furthest bin, which included non-targets located diametrically opposite to the cue (Distance bin 7 = 159°- 180°, MAD = 1.54 ± 0.01, *t*_80_ = 2.37, p = 0.020, BF_10_ = 1.70) were significantly lower than the expected value. None of the intermediate bins differed significantly from the expected MAD.

We further estimated swap error frequencies using the mixture model (fixed: 13% ± 2%, rotated: 18% ± 2%, random: 17% ± 2%) and the resultant vector method, which was applied to pooled data (fixed: 21%, rotated: 32%, random: 32%). Though the swap frequencies varied between methods of estimation, the mixture model estimates did not vary significantly between conditions (all p > 0.13, BF_10_ < 0.49).

### 4.4 Discussion

In Experiment 3 we aimed to determine whether methodological differences between previous studies in the predictability of spatial locations across trials could have influenced the frequency of swap errors, when location was used as a cue.

For fixed, rotated and random configurations, we found evidence for swap errors involving those non-target items closest to the cue location, but not for those at intermediate distances. This is consistent with results from the present and previous studies using random spatial configurations, and with the cue-feature variability account of swap errors more generally. We did not find any consistent effect of spatial predictability on swap frequency.

Additionally, in all three conditions we found that responses were on average significantly closer than expected to non-targets located precisely opposite to the cued location, suggesting a specific prevalence of confusions involving these items. This could be evidence for a non-metric representation of stimulus locations in memory, a topic we consider further in the General Discussion.

## 5 General Discussion

In cued recall tasks, observers often report features belonging to display items other than the one indicated by the cue. These erroneous responses, termed swap errors, are more likely to reflect items that are similar to the target in the cue-feature dimension (Bays, 2016a; Emrich & Ferber, 2012; Oberauer & Lin, 2017; Rerko et al., 2014; Sahan, Dalmaijer, Verguts, Husain, & Fias, 2019; Schneegans & Bays, 2017; Souza et al., 2014).

Observations analogous to swap errors have also been made in serial recall tasks commonly used in the fields of verbal and spatial short-term memory, in which participants have to recall memory items in the order they were presented. So-called transposition errors occur when participants report the correct sample items, but in an incorrect order, and most commonly take the form of swapping temporally proximate items (see Hurlstone, Hitch, & Baddeley, 2014 for review). Similarly, transposition errors are reliably observed in both immediate and delayed free recall tasks typically used to investigate memory search (Zaromb et al., 2006).

The VWM literature suggests that swap errors may be instances of a broader phenomenon reflecting the mechanism of binding items in working memory to different cue or context dimensions (Oberauer & Lin, 2017; Schneegans, McMaster, & Bays, 2022). This is consistent with broader memory literature in which transposition errors are assumed to arise from confusing contextual cues associated with each item. For example, computational models of serial recall can explicitly account for gradients of transposition errors by assuming items are bound to an evolving context built from fading records of previously encoded or retrieved items (Logan, 2021; see also Howard & Kahana, 2002). In addition, noisy coding theories (e.g., Estes, 1997) propose that transposition errors in serial recall reflect place changes between adjacent items due to the noise-driven perturbations arising from the recall of previously learned items or the study of new items. Together, in recent decades different lines of work investigating memory processes found converging empirical evidence on swap and transposition errors, as well as on the mechanistic accounts of how these errors arise.

The feature variability account proposes that swap errors are the result of confusions in identifying which item is indicated by the cue (Bays et al., 2009; Schneegans & Bays, 2017). However, some proportion of swap errors have also been ascribed to an informed guessing strategy on trials where the probed item was not in memory (Huang, 2019; Pratte, 2019), and more generally it has been suggested that at least some swap errors arise from a mechanism unrelated to cue-feature similarity (Oberauer & Lin, 2017).

To address these alternative accounts, we first assessed whether changes in variability in the cue-feature dimension induced matching changes in swap error frequency. In Experiment 1, we parametrically manipulated the precision with which stimulus orientations were encoded by varying elongation, and in Experiment 2 we manipulated encoding of stimulus motion directions by varying coherence. In each case, we tested recall performance when orientation (direction in Experiment 2) of one item was reported based on a cued location, and when location was reported based on a cued orientation (direction). Both forms of encoding manipulation were successful, as demonstrated by monotonic increases in error dispersion with decreasing elongation (coherence) when features from the manipulated dimension were reported, and an increasing concentration of responses around non-target locations when the manipulated feature dimension was used as a cue.

This latter result suggested that increasing variability in recall of orientation (direction) resulted in an increasing probability of erroneously reporting a non-target item. To assess what proportion of swap errors could be explained by this feature-variability account, we simulated responses on the location report tasks of Exps. 1 and 2 based on the errors observed on the orientation (direction) report tasks in the same experiment, using a nearest-item heuristic to estimate swap frequency. The predicted frequencies very closely matched the observed frequencies in both experiments (Fig. 4), accurately reproducing the effects of the encoding manipulations on swap frequency. This close correspondence was observed over conditions exhibiting a broad range of swap frequencies, from approximately 20% of responses in the high coherence condition of Experiment 2 to about 65% of responses in the low elongation condition of Experiment 1. These results provide evidence that variability in the cue-feature dimension is sufficient to explain swap errors in their entirety, and may be the only significant mechanism for their generation.

### 5.1 Cue-independent mechanisms

According to the feature-variability account, an erroneous report of a non-target item occurs when its cue-dimension feature retrieved from memory is more similar to the cue than the cue-dimension feature retrieved for the target. This account predicts that non-targets whose actual cue-features more closely resemble the cue (and so the target) should be reported more frequently than those less similar: a prediction that has been supported empirically (see above). However, previous work conducted by Oberauer and Lin (2017) identified a component of swap errors seemingly unrelated to cue-feature similarity. Their interference model of working memory can produce erroneous reports of non-target features through two routes. First, similar to our feature variability account, coactivation of closely located items by spatial cues may result in a non-target item being erroneously recalled. Second, background noise in the ‘context space’ can feed through to the ‘binding space’ during the delay period of the task, reactivating the report-feature representations of all items independent of their cue-feature value. The fitted parameters of their model implied that both cue-dependent and cue-independent swaps were present in their data, which came primarily from colour report tasks using a spatial cue. Furthermore, in a recently published study using the interference model, they propose that swap errors in a change-detection task were predominantly produced by cue-independent processes (Lin & Oberauer, 2022).

It is important to note that there is no simple criterion to determine whether an individual swap error was the result of confusability between a non-target item’s memorized cue feature and the given cue, or arose in a cue-independent manner. If memory precision for the cue feature is low, as is typically the case at high set sizes, then even very distant values in feature space may be confusable. The interference model makes parametric assumptions about how cue-dependent swaps are generated (specifically, a double exponential function was chosen in that model to describe the spread of activation to items similar to the cue) in order to estimate frequencies of cue-dependent and cue-independent swaps. Critically, if the parametric function chosen to describe the effect of cue-similarity is not precisely matched to the underlying generative process, swap errors that are not well-described by this distribution may instead be captured by a non-zero value for the cue-independent parameter.

In a comparison of the neural binding model with both the full interference model and a partial interference model with cue-independent activation fixed at zero, we found that the neural binding model was a consistently better fit of the data from Experiment 1 and 2. Furthermore, in Experiment 1 the partial interference model was a better fit than the full interference model which estimated cue-independent activation to be close to zero across conditions. For Experiment 2, the estimated cue-independent activation was substantially above zero in some levels of coherence in the location-report condition. The large feature separations in this experiment may increase the likelihood of the interference model attributing swap errors to cue-independent processes. However, the neural binding model remained the best fitting model overall, consistent with the feature variability account.

The neural binding model is also limited by the validity of the parametric function chosen to describe the effect of cue-similarity. While this function is to some extent validated on the basis that it is the same as the one used for report-feature variability, it could nonetheless be contested on similar grounds to the interference model. This is why we employed a non-parametric, simulation-based approach as the primary basis of our conclusions. In this approach, we only assumed that errors in memory for a feature would be distributed identically whether that feature was used as a cue or for report. Coupled with a simple cue-dependent mechanism in which the item remembered as most similar to the cue is selected for report, this proved sufficient to accurately predict the frequency of swap errors across different cue feature dimensions and reliabilities. This provides the strongest evidence against a separate cue-independent route to swap generation, because swaps generated by such a process would not be predicted on the basis of our cue-dependent method and so we would expect to observe them as a consistent underestimation of empirical swap frequency by the simulation.

We note that the relationship between cue- and report-dimension errors described above is a central assumption of the neural binding model Schneegans and Bays (2017), which is fit successfully in the current study to the data from multiple conditions simultaneously. The same assumption is not present in the interference model, which makes no particular predictions about the relationship between recall variability for a reported feature and swap errors when that same feature is used as a cue. Consequently, it was necessary to fit the interference model to each report and elongation/coherence condition separately requiring a greater number of free parameters. This is one of a number of significant differences between the models that limit the the conclusions we can draw from model comparison. A systematic factorial comparison between models may be a valuable direction for future research.

### 5.2 Effects of spatial configuration

One difference between the task designs of our Exps 1 and 2 and some previous studies, including Oberauer and Lin (2017), is that our item locations were drawn randomly and uniformly on the circle (with a minimum distance constraint), whereas in their experiments item locations were selected from a fixed set of equidistant points. It has been suggested that using a consistent fixed set of locations may reduce location noise and, as a result, influence swap frequency (Brady & Störmer, 2020; Schurgin et al., 2020). Our Experiment 3 was intended to assess this possibility.

Contrary to the proposal that using a consistent set of locations would inhibit swap errors, we found clear evidence for swaps and no significant differences in overall swap frequency when recalling stimulus arrays with fixed equidistant locations, equidistant but randomly rotated locations, or random locations. Consistent with our previous experiments, there was evidence for swap errors in Experiment 3 for non-targets closest in location to the target, in every spatial configuration (Fig. 6C). However, in each condition, we also observed a substantial dip below expected levels in the deviation of responses from non-targets positioned diametrically opposite the target, suggesting that swap errors involving these particular items were more frequent than would be predicted based on their physical (angular or Euclidean) distance from the cue.

These results could be explained by encoding of item locations in a non-metric or qualitative format (Landau & Jackendoff, 1993), in which diametrically opposing positions (180° separation on the circle) were more easily confused than some positions that were physically closer together (e.g. 120° separation). An intuitive hypothesis would be that categorical encoding would be encouraged by using a fixed set of locations across trials. However, we observed an effect of similar magnitude in the condition where locations varied from trial to trial while maintaining a regular equidistant arrangement, and of only slightly lesser magnitude in the condition where locations were generated randomly. This suggests that the opposite-item effect does not require a predictable spatial arrangement, however the effect appears confined to a narrow enough window around 180° that swaps to opposite items would be expected to occur rarely when locations are drawn at random.

We note that, despite their strict dependence on the cue, we would expect opposite-item swaps to be poorly captured by any parametric model that assumes spatial confusability declines monotonically with physical distance (e.g. Oberauer and Lin 2017; Schneegans and Bays 2017), and indeed could be mistakenly identified as having a cue-independent source.

### 5.3 Strategic accounts of swap errors

Several previous studies have argued that swap errors reflect a form of strategic behaviour, rather than a consequence of noise (Huang, 2019; Pratte, 2019; Utochkin & Brady, 2020). In particular, Pratte (2019) proposed that, when participants are instructed to recall the location of an item for which they have no memory, they may adopt a strategy of reporting a location close to where other items had been presented, rather than producing a random response. On this basis, the study concluded that the frequency of ‘guessing’ (i.e. non-target reports interpreted as guesses) in location report tasks is similar to estimates for other report feature dimensions and consistent with slot-based accounts of working memory (Cowan, 2001; Luck & Vogel, 1997; Zhang & Luck, 2008).

The hypothesis that swap errors occur when the target item is not in memory makes the clear prediction that their frequency will not depend on which feature is used as the cue. Contrary to this, and consistent with previous results of Rajsic and Wilson (2014), we observed substantially lower swap frequencies when location was the cue feature compared to when it was the report feature. Furthermore, we found that varying the reliability of the non-spatial feature (orientation or motion direction) strongly modulated swap frequencies when it was used for cueing, but had minimal effect on swap frequency when it was the reported feature. If the reliability manipulations had caused some proportion of items not to be stored, this frequency would not depend on which feature was subsequently cued for report.

The strategic guessing acount assumes a dichotomy between items that are held in memory, and those that are not, with different decision processes associated with each case. Whether this conceptualization of working memory is accurate has been a point of contention in the long-standing debate of slot versus resource models, with the latter viewing zero-information states merely as one end of a continuum of memory precisions (van den Berg et al., 2012) and recall estimates generated by a single decision process.

The feature variability account as employed here is agnostic with respect to the format and granularity of memory representations, and merely posits that the same limitations that give rise to response variability in the spatial cue condition also underlie swap errors when location is reported.

Whether a particular feature is remembered with very low precision or with zero precision makes no meaningful difference to the predictions of this account, as these cases are treated identically. Indeed, the strategy described in Pratte (2019) for guesses in spatial working memory tasks, i.e. reporting the location of whichever item’s representation in memory most closely resembles the cue, is identical to the mechanism assumed to underlie all responses in the feature variability account (Bays, 2016a; Schneegans & Bays, 2017). The difference between these proposals does not lie in the selection strategy, therefore, but in the claim that a majority of swap errors occur when the target item is not in memory. As a corollary, the previous study’s finding that participants reported non-target items when cued with a feature that wasn’t in the memory array is equally consistent with either account, and does not provide evidence for the claim that swaps are strategic guesses.

It is also worth noting that a strategy of reporting a non-target location when the cue does not match any item in memory would be highly suboptimal under a slot-based account in which only whole items can be encoded into and lost from WM: in fact, one should choose any location except those of items in memory, as those are the locations one can be certain the target did not occupy.

The neural correlates of swap errors in VWM were investigated in a recent study which found that swap error responses were preceded by active maintenance of the swapped item (Mallett, Lorenc, & Lewis-Peacock, 2022). This suggests that swap errors are not spontaneously produced random guesses at the response stage but rather memory representations for incorrect items that are maintained in VWM. Although this finding cannot rule out strategic guessing which may take place once the cue is presented but prior to the response stage, participants subjectively reported that they had a degree of confidence in their response rather than guessing.

### 5.4 Implementing the feature variability account

While not consistent with accounts in which items or slots are lost as whole units, our results could potentially be explained by accounts in which different features of an item are subject to independent variability (Bays, Wu, & Husain, 2011; Fougnie & Alvarez, 2011). To investigate this possibility, we fit the data with multiple models of VWM. Consistently the best fitting model was a previously-proposed implementation of working memory based on population coding (Schneegans & Bays, 2017). This “neural binding” model assumes that the features of stimuli are encoded in an idealized conjunctive neural code and recalled via maximum likelihood decoding from noisy neural activity. Within the model, swap errors occur when a non-target item is erroneously deemed more similar to the cue than the target (which matches the cue exactly). This process is consistent with the feature variability account of swap errors outlined previously.

The model was adapted to capture the effects of manipulating the ellipse elongation and RDK coherence by incorporating a new *conjunction coding* parameter. This addition to the model specifies a proportion of spikes that carry orientation (direction) information, in addition to the location information carried by all spikes. This reflects the expectation that stimuli with lower elongation (coherence) provide reduced orientation (direction) information, without a reduction in location information. The spiking activity resembles that of a mixed population code (Matthey, Bays, & Dayan, 2015), in which all neurons are spatially selective but only some are sensitive to orientation (direction), the activation of this latter group depending on visual discriminability of the angular feature.

The best fitting conjunction coding parameter (proportion of spikes available for orientation/direction decoding) increased significantly with increasing ellipse elongation and RDK motion coherence. The model provided a quantitatively precise account of empirical response distributions that was also parsimonious, using just six free parameters to capture full response distributions relative to both targets and non-targets across all six conditions of each experiment (i.e. all panels in Figure 3).

This idealized neural population model has a simple mathematical interpretation in terms of sampling, with each individual spike representing one noisy sample of a memorized feature value (or a combination of feature values). In this interpretation, it is intuitive that recall precision is determined by the number of spikes, or samples, that contribute to the estimation of a memorized feature. This number varies randomly and independently between items, following a Poisson process, and this stochasticity – rather than discreteness of the samples – has been shown to be critical for explaining human recall errors (Schneegans, Taylor, & Bays, 2020).

In particular, this implementation of the neural population model allows for the possibility that no samples are available for the decoding of one or more of an item’s features. Importantly this case is not dealt with differently than cases with samples available, but merely constitutes one extreme in the probabilistic distribution of recall precision (van den Berg et al., 2012). While states of strictly-zero information are not a feature of more biologically realistic population models, they have near-equivalents in states of very low precision. We tested the extent to which the zero-sample case contributed to swap errors in the model fit to the present behavioral results. While the target item had zero samples for decoding of its cue feature on a significant minority of swap trials, this proportion varied substantially between experiments and conditions. Crucially, the reported non-target item on swap trials was almost equally likely to have zero samples. This result would not be expected if observers adopted a heuristic strategy of choosing a remembered non-target when no information about the target item’s cue feature is available, but is consistent with a feature variability account in which zero-samples is simply a state of maximal variability.

More generally, the number of samples available for an individual item determines the reliability (likelihood or posterior width) of an estimate: this has been proposed as a basis for confidence judgments, accounting for trial-by-trial correlations between recall error and subjective confidence (Bays, 2016b; Fougnie, Suchow, & Alvarez, 2012; Rademaker, Tredway, & Tong, 2012; van den Berg, Yoo, & Ma, 2017). Here, we found that swap errors were more likely to occur when recall precision was low for the cue feature of both the target item and the selected non-target item. The model therefore predicts that swap trials should be associated with lower confidence ratings, explaining an empirical observation that was previously interpreted as evidence for a strategic guessing account (Pratte, 2019).

#### 5.4.1 Spatial binding and recall of multiple features

The tasks used in the present study required observers either to retrieve a non-spatial feature corresponding to a cued location, or one location associated with a cued non-spatial feature. Several previous studies have examined swap errors in contexts where one non-spatial feature is used to cue another non-spatial feature for recall (Gorgoraptis, Catalao, Bays, & Husain, 2011; Pertzov & Husain, 2014; Pertzov, Manohar, & Husain, 2017; Rajsic et al., 2017), or where observers are cued with one feature of an object to report multiple other feature dimensions (Fougnie & Alvarez, 2011; Fougnie, Cormiea, & Alvarez, 2013; Schneegans & Bays, 2017). Bays et al. (2011) found that when observers were asked to report two non-spatial features of an object on the basis of a location cue, swap errors occurred largely independently in the two reported features. This was initially interpreted as evidence against a feature variability account of swap errors, on the assumption that if variability in memory for location caused the wrong item to be identified as matching the cue, both non-spatial features of that item would be selected for report. However, subsequent investigation led to an alternative explanation for those results, compatible with the feature variability account, in which independent representations exist for the conjunction of each non-spatial feature dimension with spatial location. Because feature variability in these different representations is independent, a swap error in retrieving one non-spatial feature of an item can be accompanied by correct retrieval of a second non-spatial feature based on the same location cue. This spatial-binding model successfully accounts for the earlier results as well as a range of other tasks in which spatial and non-spatial features are used in different combinations for cueing and report (Kovacs & Harris, 2019; Schneegans & Bays, 2017, 2019).

### 5.5 Conclusion

We showed in two experiments that manipulating the reliability of a non-spatial feature in cued recall tasks produces consistent effects on recall precision (when the feature is reported) and swap probability (when the feature is used as cue). The effect on swap probability was explained in full by a feature variability account of swap errors, attributing them to variability in decoding memorized cue features, and did not require alternative explanations such as guessing strategies. In a third experiment using spatial cues, we observed a specific tendency to swap features of diametrically opposite items. While compatible with the feature variability account, this finding is not predicted by feature variability based models that assume a monotonic decrease in confusability with physical separation in space, and may contribute to discrepant findings regarding the sources of swap errors in different cued recall experiments.

## Supplementary Material

Appendices

## Figures and Tables

**Table 1:** Model fit statistics for Experiment 1. N (Param) displays the number of free parameters within each model. Δ indicates the difference in model fit statistic (AIC or BIC), averaged across participants, between each model and the best fitting model. N (Subj) indicates for how many participants each model was the best fitting model, as determined using AIC and BIC respectively.

		AIC		BIC	
Model	N (Param)	Δ	N (Subj)	Δ	N (Subj)
Neural binding model	6	0.00	10	0.00	10
Interference model (IM)	36	53.75	0	175.80	0
IM (A_a_ fixed at zero)	30	43.14	0	140.78	0

**Table 2:** Model fit statistics for Experiment 2. N (Param) displays the number of free parameters within each model. Δ indicates the difference in model fit statistic (AIC or BIC), averaged across participants, between each model and the best fitting model. N (Subj) indicates for how many participants each model was the best fitting model, as determined using AIC and BIC respectively.

		AIC		BIC	
Model	N (Param)	Δ	N (Subj)	Δ	N (Subj)
Neural binding model	6	0.52	9	0.00	10
Interference model (IM)	36	20.28	1	141.82	0
IM (A_a_ fixed at zero)	30	36.65	0	133.77	0
