# Supplementary material for: Swap errors in visual working memory are fully explained by cue-feature variability
Source: Cogn Psychol. Author manuscript; Available in PMC 2022 Jul 17. (PMC7613075; doi:10.1016/j.cogpsych.2022.101493)
Supplement: Appendices [file EMS150037-supplement-Appendices.pdf]

Appendix A

Neural binding model

The neural binding model assumes that the features of stimuli, such as their location, orientation or direction of motion, are encoded in an idealized conjunctive population code. Recall is modelled as maximum likelihood decoding from noisy neural activity. The mean firing rate of neuron k encoding cue feature ψ_j and report feature θ_j of item j in the memory array is given as

$$\bar{r}_{k,j}(\psi_j, \theta_j) = \frac{\gamma}{NM}\phi_\circ(\psi_j; \psi'_k, \kappa_\psi)\phi_\circ(\theta_j; \theta'_k, \kappa_\theta) \tag{A1}$$

Within Eq. 1, γ is the population's mean total firing rate, which is normalised over the number of items in the memory array N, and the number of neurons M involved in encoding each item. Von Mises functions with concentrations parameters κ_ψ and κ_θ for the cue and report feature and preferred values ψ'_k and θ'_k reflect the feature tuning of the neuron.

Discrete spikes are produced based on each neuron's firing rate via independent Poisson processes,

$$r_{k,j} \sim \text{Pois}(\bar{r}_{k,j}) \tag{A2}$$

Due to the superposition property of the Poisson distribution, the total number of spikes, n_j, that contribute to representing the features of each item j is then likewise a Poisson random variable,

$$n_j \sim \text{Pois}\left(\frac{\gamma}{N}\right). \tag{A3}$$

Response probabilities

Recall is modeled as maximum likelihood decoding from noisy neural activity over a fixed time window. For a given number of spikes n_j available for decoding the features of

item j, the distribution of decoded values $\hat{\theta}_j$ can be described as a von Mises distribution[1]., which is centred on the true feature value θ_j and whose precision is linearly increasing with the number of spikes, n_j:

$$p_{\text{dec}}\left(\hat{\theta}_j|\theta_j, n_j\right) = \phi_{\circ}(\hat{\theta}_j; \theta_j, \kappa(n_j\omega_\theta)) \tag{A4}$$

Here, ω_θ is the precision (as Fisher information) corresponding to the tuning curve concentration κ_θ, which is determined as $\omega = \kappa\frac{I_1(\kappa)}{I_0(\kappa)}$. The term $\kappa(n_j\omega_\theta)$ describes the concentration parameter yielding a von Mises distribution with precision $n_j\omega_\theta$, which can be obtained by numerical inversion of the same relationship. The joint distribution of decoded cue and report feature values can then be described as weighted sum of decoding probabilities for each possible spike count n_j,

$$p_{\text{dec}}\left(\hat{\theta}_j, \hat{\psi}_j|\theta_j, \psi_j\right) = \sum_{n_j=0}^{\infty} \Pr_{\text{Pois}}\left(n_j, \frac{\gamma}{N}\right) p_{\text{dec}}(\hat{\theta}_j|\theta_j, n_j) p_{\text{dec}}(\hat{\psi}_j|\psi_j, n_j). \tag{A5}$$

As both feature dimensions depend on the same number of spikes, the decoding errors in each dimension are not independent. The model assumes that cue and report features of all items within the memory array are decoded upon presentation of the cue. The item with the closest decoded feature to the cue feature would then be used to produce the response (of the item's report feature). The probability that a certain report feature value θ_r is chosen as a response in a trial with item report and cue feature values $\boldsymbol{\theta}$ and $\boldsymbol{\psi}$, respectively, is then

$$p_{\text{resp}}\left(\theta_r|\boldsymbol{\theta}, \boldsymbol{\psi}\right) = \sum_{j=1}^{N} p(\hat{\theta}_j = \theta_r \wedge \text{item } j \text{ selected } |\boldsymbol{\theta}, \boldsymbol{\psi}). \tag{A6}$$

The probability that an item is selected for response generation is determined by its decoded cue feature, and due to the aforementioned dependence between decoding errors it

[1] Note that this is not an exact solution for the maximum likelihood decoding in circular feature spaces, but it provides a close approximation within the range of typical parameter values of the neural population model (Schneegans et al., 2020)

is not independent from the obtained report feature value. But we can separate these probabilities by conditioning on the number of available spikes, n_j :

$$p_{\text{resp}}\left(\theta_r|\boldsymbol{\theta},\boldsymbol{\psi}\right)=\sum_{j=1}^{N}\sum_{n_j=0}^{\infty}\text{Pr}_{\text{Pois}}\left(n_j,\frac{\gamma}{N}\right)p_{\text{dec}}(\theta_r|\theta_j,n_j)\text{Pr}_{\text{sel}}(j|\boldsymbol{\psi},n_j) \tag{A7}$$

The conditional probability of decoding a certain report feature value given the spike count and true feature value in this equation can be determined as in Eq. 4. The probability that an item is selected (i.e., its decoded cue feature value is closest to the actual cue) can be computed by numerical integration as

$$\text{Pr}_{\text{sel}}\left(j|\boldsymbol{\psi},n_j\right)=\int_0^{\pi}p(D_{\circ}(\hat{\psi}_j-\psi_c)=s|\psi_j,n_j)\prod_{j'\neq j}p(D_{\circ}(\hat{\psi}_{j'}-\psi_c)>s|\psi_{j'})ds, \tag{A8}$$

where ψ_c is the feature value of the actually given cue. The first probability term in this integral can be evaluated based on Eq. 4, while the second term requires marginalizing over the possible sample counts,

$$p_{\text{dec}}\left(\hat{\psi}_j|\psi_j\right)=\sum_{n_j=0}^{\infty}\text{Pr}_{\text{Pois}}(n_j,\frac{\gamma}{N})p_{\text{dec}}(\hat{\psi}_j|\psi_j,n_j). \tag{A9}$$

Conjunction coding parameter

The model was adapted in order to capture the effect of varying the ellipse elongation and the motion coherence of the RDK on memory for orientation and motion direction respectively. Specifically, the number of spikes that contribute to orientation or direction decoding, irrespective of whether it is the cue or report feature, was allowed to vary from the number of spikes contributing to location decoding. An analogous modification of this model has been employed previously to capture specific impairments in feature binding or report precision following stroke (Lugtmeijer et al., 2021).

The parameter s was added to the model, which specifies the mean proportion of the total spikes, n_j, that are available for decoding an item's orientation or direction value,

respectively. The adjusted number of spikes was assumed to be drawn from a binomial distribution with success rate s. If the feature with reduced number of spikes was used as cue feature in the task under consideration, the selection probability (used in Eq. A7) is adjusted to

$$\text{Pr}_{\text{sel}}\left(j|\boldsymbol{\psi}, n_j, s\right) = \sum_{\tilde{n}_j=0}^{n_j} \text{Pr}_{\text{Binom}}(\tilde{n}_j; n_j, s)\text{Pr}_{\text{sel}}(j|\boldsymbol{\psi}, \tilde{n}_j), \tag{A10}$$

If the feature with reduced number of spikes contributing to its decoding is the report feature, the decoding probability (likewise used in Eq. A7) has to be adjusted, yielding

$$p_{\text{dec}}\left(\theta|\theta_j, n_j, s\right) = \sum_{\tilde{n}_j=0}^{n_j} \text{Pr}_{\text{Binom}}(\tilde{n}_j; n_j, s)p_{\text{dec}}(\theta|\theta_j, \tilde{n}_j). \tag{A11}$$

Estimating spike count for swap trials

The probability that the item which is selected for response generation has a certain associated spike count, n_j, can be determined via Bayes' theorem from the conditional probability that the item is selected if it has that spike count:

$$\text{Pr}(n_j| \text{ item } j \text{ selected }) \propto \text{Pr}_{\text{sel}}\left(j|\boldsymbol{\psi}, n_j\right)\text{Pr}(n_j) \tag{A12}$$

The selection probability is given in Eq. A8, and the a-priori probability for n_j follows a Poisson distribution, as given in Eq. A3. If the decoded feature has a reduced number of spikes available due to the conjunction coding parameter s, the conditional selection probability is instead given by Eq. A10.

To obtain the conditional probability that the selected item in a trial has a spike count n_j given that a swap error occurred in that trial, we compute the sum over the probabilities given in Eq. A12 for all non-target items, weighted with the probabilities that each non-target item is selected (Eq. A8 or A10).

Appendix B

Model fitting

Neural binding model

Maximum likelihood fits of the model were obtained for the data of each participant, at each level of ellipse elongation (Experiment 1) or RDK motion coherence (Experiment 2). The model applied six free parameters. The widths of the von Mises tuning curves for orientation or direction, and location (initial value: location = 8, orientation = 3, direction = 3), the mean total spike rate in the neural population (initial value: 8) and the conjunction coding parameter for each level of elongation or motion coherence (initial value: 0.5).

A hierarchical optimisation approach was applied in order to avoid local minima. This involved running an initial maximum likelihood fit to determine the best fitting tuning curve and mean total spike rate parameters. Then, for each iteration of these parameter values applied in the search, a secondary fit was carried out to determine the best fitting conjunction coding parameter for each condition.

For all fits, we set an upper limit for the gain parameter γ (mean total number of spikes in the neural population). In the model, there is a possible trade-off between tuning curve widths and gain parameter, which jointly determine the decoding precision. If the model is fit to behavioral data with different set sizes, the model's assumption of a fixed total spike rate that is distributed across all items in a trial is typically sufficient to constrain estimates for all parameters. However, since only a single set size is used in the present study, the optimization procedure to determine parameter values can produce an unbounded increase in the gain parameter (and concurrent decrease of the tuning curve widths), without significant changes in the resulting decoding distributions. The limit on γ was set to 40 for all model fits, based on typical parameter ranges observed in previous fits to similar experiments (Schneegans & Bays, 2017).

Interference model

The interference model was fit to the data using a range of initial values for each free parameter. The six free parameters in the model were the precision of memory for items when they are outside of the focus of attention (κ; initial values: 4, 8, 15), the precision of the one item within the focus of attention (κ_{f}; initial values: 5, 20, 40), the weights of the cue-independent retrieval component (A_a; initial values: 0.01, 0.2, 0.5, 1.0) and, background noise (A_b; initial values: 0.01, 0.2, 0.5, 1.0), the width of the exponential function that determines item selection in the cue-based retrieval (s; initial values: 0.1, 1, 5, 10), and finally the proportional reduction of weights A_a and A_b when the target item is in the focus of attention (r; initial values: 0.1, 0.4, 0.8).

The partial model used the same initial values for each parameter, as specified above, apart from context-independent activation ($\mathrm{A_a}$) which was fixed at zero. Once parameter estimates were obtained for the partial model, the full model was fit to the data again using the parameter estimates from the partial model as initial values ($\mathrm{A_a}$ initial value: 0.01). The full model with the greatest maximum likelihood was used in the model comparison.

Table A1

Neural binding model parameters (Experiment 1). Group mean and SE values for mean total firing rate in the neural population (γ), tuning curve widths for orientation (κ_{ori}) and location (κ_{loc}) as well as the conjunctive coding parameters for the low (s_{low}), medium (s_{med}) and high (s_{high}) elongation conditions.

	γ	κ_{ori}	κ_{loc}	s_{low}	s_{med}	s_{high}
Mean	34.70	1.40	3.54	0.30	0.46	0.67
SE	3.02	0.15	0.44	0.05	0.10	0.11

Table A2

Interference model parameters (Experiment 1). Group mean values (SE in parentheses) for the relative weights of the context-independent (A_a) and background noise (A_b) activation, the proportional reduction of interference within the focus of attention r, the steepness of the cue-based retrieval function (s), and the precision of representations outside (κ) and inside (κ_f) the focus of attention.

Model	A_a	A_b	r	s	κ	κ_f
**Interference model (IM)**						
Ori cue, Low	0.19	0.07	0.50	0.86	16.85	89.88
	(0.19)	(0.04)	(0.09)	(0.12)	(2.43)	(31.10)
Ori cue, Medium	0.05	0.00	0.35	1.15	19.28	6.62
	(0.04)	(0.00)	(0.11)	(0.14)	(3.86)	(2.24)
Ori cue, High	0.02	0.02	0.43	1.52	20.88	18.50
	(0.01)	(0.01)	(0.09)	(0.18)	(3.36)	(5.64)
Loc cue, Low	0.05	0.05	0.34	8.51	2.59	1312.77
	(0.05)	(0.04)	(0.09)	(3.12)	(0.49)	(1304.64)
Loc cue, Medium	0.02	0.04	0.34	12.32	3.66	82.29
	(0.01)	(0.03)	(0.10)	(3.83)	(1.09)	(62.64)
Loc cue, High	0.04	0.02	0.29	20.47	4.13	14.32
	(0.03)	(0.01)	(0.08)	(6.96)	(0.87)	(5.70)
**IM (A_a fixed at zero)**						
Ori cue, Low	0	0.03	0.40	0.79	15.63	63.94
		(0.03)	(0.11)	(0.13)	(2.65)	(23.12)
Ori cue, Medium	0	0.00	0.37	1.04	18.65	11.78
		(0.00)	(0.10)	(0.15)	(3.69)	(3.64)
Ori cue, High	0	0.02	0.34	1.46	20.86	18.32
		(0.02)	(0.08)	(0.17)	(3.35)	(6.00)
Loc cue, Low	0	0.01	0.33	9.49	2.24	14.66
		(0.00)	(0.09)	(5.85)	(0.34)	(9.05)
Loc cue, Medium	0	0.04	0.54	17.76	3.73	78.04
		(0.03)	(0.09)	(7.02)	(1.07)	(56.93)
Loc cue, High	0	0.20	0.37	23.31	9.22	14.64
		(0.17)	(0.07)	(7.48)	(4.87)	(5.72)

Table A3

Neural binding model parameters (Experiment 2). Group mean and SE values for mean total firing rate in the neural population (γ), tuning curve widths for motion direction (κ_{dir}) and location (κ_{loc}) as well as the conjunctive coding parameters for the low (s_{low}), medium (s_{med}) and high (s_{high}) coherence conditions.

	γ	κ_{dir}	κ_{loc}	s_{low}	s_{med}	s_{high}
Mean	28.55	6.55	4.45	0.06	0.10	0.26
SE	4.04	0.84	0.50	0.01	0.02	0.04

Table A4

Interference model parameters (Experiment 2). Group mean values (SE in parentheses) for the relative weights of the context-independent (A_a) and background noise (A_b) activation, the proportional reduction of interference within the focus of attention r, the steepness of the cue-based retrieval function (s), and the precision of representations outside (κ) and inside (κ_f) the focus of attention.

Model	A_a	A_b	r	s	κ	κ_f
**Interference model (IM)**						
Dir cue, Low	0.82	0.06	0.19	8.13	24.50	120.58
	(0.16)	(0.05)	(0.04)	(3.00)	(4.12)	(64.39)
Dir cue, Medium	0.62	0.08	0.37	9.37	25.48	33.09
	(0.24)	(0.07)	(0.10)	(2.35)	(2.52)	(16.96)
Dir cue, High	0.13	0.01	0.17	7.64	23.92	35.23
	(0.05)	(0.00)	(0.05)	(2.66)	(3.32)	(10.12)
Loc cue, Low	0.04	0.55	0.39	8.64	7.02	150.55
	(0.02)	(0.13)	(0.10)	(3.01)	(1.58)	(76.15)
Loc cue, Medium	0.03	0.20	0.33	12.31	11.70	13.49
	(0.01)	(0.08)	(0.07)	(4.06)	(2.51)	(6.21)
Loc cue, High	0.19	0.03	0.37	14.30	7.79	93.33
	(0.16)	(0.02)	(0.10)	(4.02)	(0.88)	(60.59)
**IM (A_a fixed at zero)**						
Dir cue, Low	0	0.06	0.15	0.25	23.27	55.64
		(0.05)	(0.07)	(0.05)	(3.12)	(25.55)
Dir cue, Medium	0	0.03	0.29	0.76	24.44	46.08
		(0.01)	(0.05)	(0.28)	(2.73)	(21.33)
Dir cue, High	0	0.03	0.51	1.89	23.53	49.99
		(0.01)	(0.11)	(0.35)	(3.19)	(10.55)
Loc cue, Low	0	0.64	0.40	8.82	10.95	74.56
		(0.16)	(0.09)	(3.23)	(4.16)	(44.88)
Loc cue, Medium	0	0.22	0.44	11.30	11.53	24.22
		(0.08)	(0.11)	(3.68)	(2.58)	(11.21)
Loc cue, High	0	0.05	0.32	14.44	8.07	122.83
		(0.03)	(0.05)	(4.29)	(0.95)	(82.25)

Table A5

Group mean cue-feature memory precision, of the target and selected non-target item, in target or swap error trials (mean ±1 SE across participants).

	Target trials	Swap error trials	
Condition	Target item	Target item	Selected item
Experiment 1			
Orientation cue			
Low	1.59 (0.33)	1.09 (0.22)	1.20 (0.25)
Medium	2.23 (0.45)	1.69 (0.35)	1.82 (0.38)
High	3.21 (0.60)	2.61 (0.49)	2.78 (0.53)
Location cue			
Low	17.84 (3.09)	13.23 (2.18)	14.68 (2.46)
Medium	17.68 (3.08)	13.20 (2.15)	14.52 (2.42)
High	17.83 (3.09)	13.44 (2.21)	14.52 (2.38)
Experiment 2			
Direction cue			
Low	4.02 (0.64)	0.70 (0.11)	1.24 (0.22)
Medium	6.67 (0.99)	1.40 (0.23)	2.90 (0.56)
High	11.96 (2.01)	2.72 (0.42)	7.37 (1.49)
Location cue			
Low	24.59 (3.35)	9.06 (1.15)	18.15 (2.79)
Medium	24.56 (3.35)	8.90 (1.88)	18.44 (3.60)
High	24.56 (3.36)	9.91 (1.48)	18.61 (4.21)

Table A6

Group mean report-feature memory precision, of the target and selected non-target item, in target or swap error trials (mean ±1 SE across participants).

	Target trials	Swap error trials	
Condition	Target item	Target item	Selected item
Experiment 1			
Orientation cue			
Low	18.55 (3.27)	16.70 (2.94)	17.10 (3.02)
Medium	18.30 (3.23)	16.35 (2.89)	16.80 (2.98)
High	18.37 (3.20)	16.23 (2.87)	16.84 (2.98)
Location cue			
Low	1.35 (0.28)	1.00 (0.22)	1.11 (0.24)
Medium	2.01 (0.42)	1.53 (0.33)	1.67 (0.35)
High	3.03 (0.58)	2.33 (0.46)	2.51 (0.49)
Experiment 2			
Direction cue			
Low	25.38 (3.45)	23.56 (3.38)	23.85 (3.39)
Medium	25.40 (3.51)	22.70 (3.29)	23.43 (3.33)
High	25.20 (3.45)	20.24 (3.04)	22.67 (3.29)
Location cue			
Low	2.25 (0.39)	0.96 (0.26)	1.59 (0.23)
Medium	4.79 (0.83)	2.05 (0.63)	2.77 (0.56)
High	10.67 (1.98)	4.89 (1.66)	7.41 (1.34)

Table A7

The conditional probability that the target or selected non-target item received zero samples in the cue-feature dimension in target or swap error trials (median % and interquartile range across participants).

	Target trials	Swap error trials	
Condition	Target item	Target item	Selected item
Experiment 1			
Orientation cue			
Low	7.52 (3.54-26.84)	31.42 (11.39-55.76)	25.74 (9.58-51.36)
Medium	2.95 (1.13-20.67)	13.17 (5.20-48.43)	10.63 (4.19-43.67)
High	0.48 (0.08-8.77)	3.17 (0.48-29.07)	2.16 (0.36-24.12)
Location cue			
Low	0.03 (0.03-0.11)	2.90 (2.46-7.45)	0.98 (0.68-2.71)
Medium	0.04 (0.03-0.12)	2.71 (2.43-7.41)	0.89 (0.73-3.35)
High	0.04 (0.03-0.10)	2.78 (1.78-7.26)	1.05 (0.66-3.47)
Experiment 2			
Direction cue			
Low	44.34 (43.35-51.42)	88.64 (87.51-90.80)	82.14 (79.19-83.25)
Medium	27.80 (18.08-33.63)	83.06 (80.34-87.88)	70.22 (59.59-76.71)
High	6.59 (5.32-12.29)	74.65 (67.40-76.44)	39.22 (36.03-50.59)
Location cue			
Low	0.07 (0.00-0.44)	44.32 (11.82-56.54)	8.88 (0.82-19.66)
Medium	0.07 (0.00-0.48)	53.15 (13.23-61.98)	5.34 (0.54-19.81)
High	0.06 (0.00-0.50)	48.46 (9.52-55.27)	14.74 (0.00-19.21)

Table A8

The conditional probability that the target or selected non-target item received zero samples in the report-feature dimension in target or swap error trials (median % and interquartile range across participants).

	Target trials	Swap error trials	
Condition	Target item	Target item	Selected item
Experiment 1			
Orientation cue			
Low	0.06 (0.05-0.18)	0.22 (0.18-0.63)	0.18 (0.17-0.56)
Medium	0.05 (0.04-0.16)	0.28 (0.21-0.76)	0.18 (0.17-0.64)
High	0.05 (0.04-0.14)	0.32 (0.27-0.79)	0.23 (0.20-0.62)
Location cue			
Low	19.11 (7.28-42.13)	30.40 (21.92-57.97)	26.12 (14.51-52.42)
Medium	7.11 (2.79-32.89)	16.47 (10.84-45.34)	11.69 (6.74-42.36)
High	1.22 (0.06-14.05)	9.04 (3.71-31.15)	4.66 (1.47-26.55)
Experiment 2			
Direction cue			
Low	0.10 (0.00-0.79)	0.20 (0.01-1.51)	0.19 (0.00-1.40)
Medium	0.08 (0.00-0.67)	0.22 (0.01-1.80)	0.20 (0.01-1.53)
High	0.08 (0.00-0.56)	0.29 (0.03-3.72)	0.19 (0.01-2.13)
Location cue			
Low	68.89 (68.16-72.95)	88.83 (77.96-91.76)	79.35 (74.96-81.28)
Medium	50.20 (36.84-58.13)	82.40 (53.86-92.61)	67.80 (51.71-75.69)
High	15.09 (11.48-26.53)	63.19 (34.29-84.36)	37.89 (33.32-48.50)
